# Gageostatins A–C, Antimicrobial Linear Lipopeptides from a Marine *Bacillus subtilis*

**DOI:** 10.3390/md12020871

**Published:** 2014-01-31

**Authors:** Fakir Shahidullah Tareq, Min Ah Lee, Hyi-Seung Lee, Jong-Seok Lee, Yeon-Ju Lee, Hee Jae Shin

**Affiliations:** 1 Department of Marine Biotechnology, University of Science and Technology, 176 Gajung-dong, 217 Gajungro Yuseong-gu, Daejeon, 305-350, Korea; E-Mail: stareqfakir@gmail.com; 2 Marine Natural Products Chemistry Laboratory, Korea Institute of Ocean Science and Technology, Ansan, 426-744, Korea; E-Mails: yimina82@kiost.ac (M.A.L.); hslee@kiost.ac (H.-S.L.); jslee@kiost.ac (J.-S.L.); yjlee@kiost.ac (Y.-J.L.)

**Keywords:** *Bacillus subtilis*, lipopeptides, antimicrobial activity, cytotoxicity

## Abstract

Concerning the requirements of effective drug candidates to combat against high rising multidrug resistant pathogens, we isolated three new linear lipopeptides, gageostatins A–C (**1**–**3**), consisting of hepta-peptides and new 3-β-hydroxy fatty acids from the fermentation broth of a marine-derived bacterium *Bacillus subtilis*. Their structures were elucidated by analyzing a combination of extensive 1D, 2D NMR spectroscopic data and high resolution ESIMS data. Fatty acids, namely 3-β-hydroxy-11-methyltridecanoic and 3-β-hydroxy-9,11-dimethyltridecanoic acids were characterized in lipopeptides **1 **and **2, **respectively, whereas an unsaturated fatty acid (*E*)-7,9-dimethylundec-2-enoic acid was assigned in **3**. The 3*R* configuration of the stereocenter of 3-β-hydroxy fatty acids in **1** and **2** was established by Mosher’s MTPA method. The absolute stereochemistry of amino acid residues in **1**–**3** was ascertained by acid hydrolysis followed by Marfey’s derivatization studies. Gageostatins **1**–**3** exhibited good antifungal activities with MICs values of 4–32 µg/mL when tested against pathogenic fungi (*R. solani*, *B. cinerea* and *C. acutatum*) and moderate antibacterial activity against bacteria (*B. subtilis,* S. *aeureus, S. typhi and P. aeruginosa*) with MICs values of 8–64 µg/mL. Futhermore, gageostatins **1**–**3 **displayed cytotoxicity against six human cancer cell lines with GI_50_ values of 4.6–19.6 µg/mL. It is also noteworthy that mixed compounds **1**+**2** displayed better antifungal and cytotoxic activities than individuals.

## 1. Introduction

Marine ecosystems comprise a productive and enormous resource of vast chemical entities with immeasurable biological activities [1]. Given such a background, the chemistry of marine natural products has been progressing at an unprecedented rate, resulting in discoveries of a wide range of carbon skeletons and molecules that are not usually found from terrestrial sources [2]. In fact, marine microorganisms are ubiquitous in the marine environment, and can tolerate adverse conditions such as high temperature, pressure, salinity and pH, and possess unique metabolic pathways that are different from their terrestrial counterparts [3]. *Bacillus subtilis* is an example of widely distributed bacterium, capable of growth in many environments, and exhibits considerable genomic diversity [4]. A genomic study on the widely distributed *Bacillus* strains revealed that about eight percent of its genome is devoted to synthesizing antibiotics [5,6]; whereas, a genomic study on a marine-derived *B. subtilis* subsp. *spizisenii* strain gtP20b, isolated from the Indian Ocean, revealed the presence of huge number of genes for biosynthesis of secondary metabolites [7]. As a consequence, a large number of natural product research on marine-derived *Bacillus* sp. resulted in the discovery of a diverse class of secondary metabolites including lipopeptides, polypeptides, macrolactones, fatty acids, polyketides, lipoamides, and isocoumarins [8]. Among these compounds, lipopeptides (LPs) of the surfactin, iturin, and fengycin families have been found to exhibit a wide range of bioactivities including antimicrobial, antiviral, anticancer, immunosuppressive, antituberculosis, antimycoplasmic and exceptional surfactant properties [9,10,11]. 

LPs are nonribosomal oligopeptides produced by large multienzyme complexes in several *Bacillus* species and share a common cyclic structure including a β*-*amino or β-hydroxyl fatty acid that is integrated into the peptide moiety [12]. The mode of action of some of them is via perturbation of the cell membrane of the microorganism, thus affecting the transmembrane electric potential [13]. The major step involves the binding of cationic LPs to the negatively charged lipopolysaccharide of Gram-negative bacteria or to the lipotechoic acid of Gram-positive bacteria [14,15]. In fungi, the LPs bind to the negatively charged membrane phosphatidylinositol and to the negatively charged terminal sialic acid moieties [16,17]. For example, echinocandins and pneumocandins display strong antifungal activities by inhibiting the synthesis of 1,3-β-d-glucan, an essential cell wall homopolysaccharide in pathogenic fungi including *Aspergillus* and *Candida* [18]. 

**Figure 1:**
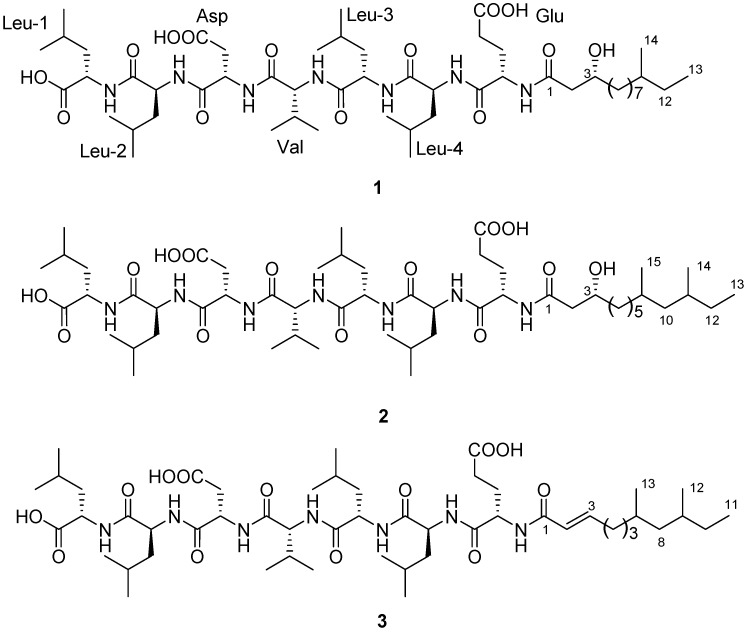
Structures of Gageosatins A–C (**1**–**3**).

As the multidrug-resistant pathogens are emerging day by day with regularity, it has now become an urgent issue to discover new antimicrobials to combat against them. Concerning the requirements for the new antibiotics, we have focused our attention on the isolation of antimicrobial secondary metabolites from the bacterium *B. subtilis*, whose crude extract showed potent antimicrobial activity. Chemical investigations from the fermentation broth of the bacterium *B. subtilis* yielded three new linear LPs, gageostatins A (2.8 mg), B (3.2 mg) and C (1.5 mg) (Figure 1). We report here the details of isolation, structural characterization and antimicrobial activities of these new LPs.

## 2. Results and Discussion

### 2.1. Isolation of Compounds

The bacterial strain 109GGC020 was isolated from a marine sediment sample collected from Gageocho, in the Republic of Korea’s southern reef, and identified as *B. subtilis* by 16s rRNA sequencing. As the physiological process of bacteria varies with the variation of surrounding living environment and metabolic profiles can be changed with very small changes in cultivation conditions [19,20] the growth conditions of the strain for maximum metabolites production were optimized through culturing in combination of different salinity of water, pH, and temperature before proceeding of large scale culture. The optimal salinity of water, pH and temperature were found to be 18.3 g/L, 7.02 and 24 °C, respectively, for the maximum growth of the strain. The strain was then cultured following above conditions and the fermentation broth was extracted with EtOAc. Thereafter, three new linear lipopeptdes gageostatins A–C (**1**–**3**), were isolated from the EtOAc extract by sequential fractionations and purifications employing flash column chromatography followed by reversed phase HPLC techniques. 

### 2.2. Structure Determination

Gageostatin A (**1**) was isolated as an amorphous solid and showed a molecular ion peak at *m/z* 1062.6691 [M + Na]^+^ in the HR-ESIMS spectrum corresponding to the molecular formula of C_52_H_93_N_7_O_14_ (see Supplementary Information). The IR spectra of **1** gave prominent broad peaks at 3291 cm^−1^ (NH) and 1646–1737 cm^–1^ (CO), consistent with the presence of amide carbonyl groups and a broad peak at 2930 cm^−1^ confirmed the presence of aliphatic chain. The ^1^H NMR data (Table 1), recorded in both CD_3_OD and CD_3_OH of the chromatographically homogeneous material, revealed the presence of a long aliphatic chain (CH_2_ at δ_H_ 1.29) and a peptide backbone by 7 NH signals at δ_H_ 7.68–8.79 together with α-protons between δ_H_ 4.13 and 4.57. A resonance at δ_H_ 3.98 indicated the presence of a further oxygenated proton, and CH_3_ signals were observed at δ_H_ 0.86–0.98. Furthermore, the ^13^C NMR spectrum of **1 **indicated the presence of 10 carbonyl carbons at δ_C _173.6–181.5, which were attributable to amino acids. These detailed IR absorbencies together with ^1^H and ^13^C data analysis indicated the lipopeptidic nature of **1**. 

**Table 1:** ^1^H and ^13^C NMR and HMBC data of **1**–**3** in CD_3_OD.

		1		2		3	
Units	No.	δ_H_, mult. (*J* in Hz)	δ_C_	δ_H_, mult. (*J* in Hz)	δ_C_	δ_H_, mult. (*J* in Hz)	δ_C_
Leu-1							
	1		180.3		180.2		180.2
	2	4.37 m	53.5	4.37 m	53.3	4.30 m	54.8
	3	1.65 m	41.6	1.65 m	41.6	1.63 m	41.6
	4	1.65 m	26.1	1.65 m	26.1	1.63 m	26.1
	5	0.91 m	21.7	0.91 m	21.7	0.89 m	21.8
	6	0.91 m	23.5	0.91 m	23.4	0.89 m	23.4
	NH	8.37 d (8.0) ^a^		8.37 d (8.0) ^a^		8.52 d (9.5) ^a^	
Leu-2							
	1		173.6		173.5		175.7
	2	4.38 m	53.4	4.38 m	53.3	4.39 m	53.3
	3	1.65 m	43.4	1.65 m	43.4	1.63 m	41.4
	4	1.65 m	26.1	1.65 m	26.1	1.63 m	26.1
	5	0.91 m	21.7	0.91 m	21.7	0.96 m	22.3
	6	0.91 m	23.6	0.91 m	23.6	0.96 m	24.0
	NH	7.96 d (8.0) ^a^		7.97 d (8.0) ^a^		7.67 d (9.0) ^a^	
Asp							
	1		174.3		174.2		174.3
	2	4.57 t (6.0)	53.1	4.57 m	53.0	4.56 m	53.2
	3	2.56 dd (16.5, 5.5)	40.1	2.56 dd (16.5, 5.0)	40.0	2.51 dd (16.5, 5.5)	40.1
		2.76 dd (16.5, 6.0)		2.75 dd (16.5, 5.5)		2.75 dd (16.5, 6.0)	
	NH	8.38 d (8.0) ^a^		8.39 d (8.0) ^a^		8.40 d (9.0) ^a^	
	COOH		178.2		178.0		178.2
Val							
	1		173.5		173.5		173.7
	2	4.13 d (8.0)	61.0	4.13 m	60.9	4.06 m	61.2
	3	2.18 m	31.2	2.17 m	31.2	2.13 m	31.2
	4	0.93 m	19.3	0.93 m	19.7	0.96 m	19.8
	5	0.93 m	20.2	0.93 m	20.0	0.96 m	20.1
	NH	7.96 d (8.0) ^a^		7.97 d (8.0) ^a^		7.97 d (8.0) ^a^	
Leu-3							
	1		175.0		174.9		175.5
	2	4.40 m	54.8	4.40 m	54.7	4.35 m	53.5
	3	1.65 m	41.4	1.65 m	41.4	1.65 m	40.4
	4	1.65 m	26.1	1.65 m	26.1	1.63 m	26.1
	5	0.93 m	22.2	0.93 m	22.1	0.92 m	22.4
	6	0.93 m	23.9	0.93 m	23.8	0.92 m	23.8
	NH	7.68 d (9.0) ^a^		7.68 d (9.0) ^a^		7.96 d (8.0) ^a^	
Leu-4							
	1		175.6		175.5		173.6
	2	4.31 m	54.2	4.31 m	54.1	4.33 m	55.5
	3	1.65 m	41.3	1.65 m	41.3	1.63 m	40.4
	4	1.65 m	26.1	1.65 m	26.1	1.63 m	26.1
	5	0.93 m	22.3	0.93 m	22.2	0.92 m	22.3
	6	0.93 m	24.1	0.93 m	23.9	0.92 m	23.9
	NH	8.48 d (6.5) ^a^		8.49 d (6.5) ^a^		8.51 m	
Glu							
	1		175.0		174.9		175.1
	2	4.34 m	55.5	4.34 m	55.4	4.35 m	55.5
	3	1.95 m	29.6	1.95 m	29.5	1.95 m	29.8
		2.05 m		2.05 m		2.03 m	
	4	2.29 t (7.0)	35.4	2.29 m	35.4	2.26 m	35.5
	COOH		181.5		181.4		181.5
	NH	8.79 d (6.5) ^a^		8.81 d (6.5) ^a^		8.81 brd.	
3-OH acid							
	1		175.4		175.3		169.1
	2	2.33 dd (9.0, 14.5)	45.0	2.33 dd (8.5, 14.0)	44.7	6.01 d (15.5)	124.7
		2.46 dd (4.0, 14.5)		2.47 dd (4.0, 14.5)			
	3	3.98 m	70.2	3.98 m	70.0	6.79 dt(15.5, 8.5)	146.5
	4	1.34 m	26.9	1.34 m	26.9	2.18 m	33.2
		1.48 m		1.47 m			
	5	1.49 m	38.6	1.49 m	38.5	1.45 m	29.7
	6	1.29 brs.	28.1–31.3	1.29 brs.	28.1–31.3	1.16 m	40.4
	7					1.52 m	29.3
	8			1.17 m	40.3	1.28 brds.	30.8
	9			1.54 m	29.2	1.28 brds.	35.8
	10	1.17 m	40.4	1.29 brs.	30.8	1.12 m	37.9
						1.28 m	
	11	1.51 m	29.3	1.29 brs.	35.7	0.87 m	11.9
	12	1.29 brs.	38.5	1.12 m	37.8	0.86 m	14.6
				1.29 m			
	13	0.86 m	14.6	0.87 m	11.9	0.87 m	23.2
	14	0.87 m	23.1	0.86 m	14.5		
	15			0.87 m	23.1		

^a^ ^1^H NMR data recorded in CD_3_OH.

**Figure 2:**
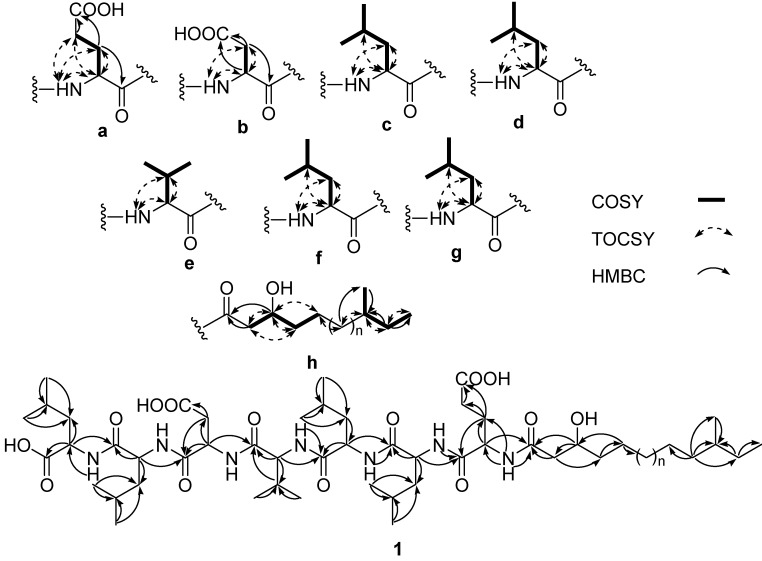
Assignment of partial structures (**a**–**h**) by COSY and TOCSY correlations and complete structure by HMBC correlations of gageostatins A.

The detailed analysis of COSY, TOCSY and HMBC correlations enabled us to identify partial substructures **a**–**h** corresponded to amino acid residues and a fatty acid moiety that constituted **1 **(Figure 2). The spin systems of the α-H and NH resonances of Glu (δ_H_ 4.34/ 8.79), Asp (δ_H_ 4.57/8.38), Val (δ_H_ 4.13/7.96) and Leu (δ_H_ 4.37/8.37, δ_H_ 4.38/7.96, δ_H_ 4.40/7.68, δ_H_ 4.37/8.48), and the correlations from the α-methine resonances to the side chains of the individual amino acid residues were observed in the COSY spectrum. Excitation of the amide proton signal at δ_H_ 8.79 showing TOCSY correlations with α-methine at δ_H_ 4.34 and methylene signals at δ_H_ 1.95 and 2.05, in addition, α-methine signals showing correlations with methylene signals at δ_H_ 1.95/2.05 and 2.29, revealed the presence of a glutamic acid unit. Correlations observed from the methylene protons at δ_H_ 1.95/2.05 and 2.29 to a carboxylic carbon resonated at δ_C_ 181.5 and the protons at δ_H_ 1.95/2.05 to carbonyl carbon at δ_C_ 175.0 in a HMBC spectrum confirmed the glutamic acid as a substructure “**a**”. Likewise, an amide proton signal at δ_H_ 8.38 showed connectivities with methine and methylene protons resonated at δ_H_ 4.57 and 2.56/2.76 in TOCSY experiment, further connectivity of these protons with carbonyl and carboxylic carbons at δ_C_ 174.3 and 178.2 observed in HMBC experiment revealed the presence of Asp unit (substructure **b**). In an entirely analogous way, substructures **c**–**g **(only COSY and TOCSY correlations are shown in Figure 2) were assigned by using 1D and 2D NMR data. Moreover, the TOCSY and HMBC spectra allowed identifying a 3-β-hydroxyl fatty acid residue in the molecule **1 **as a substructure **h**. In the ^13^C NMR spectra of **1**, there were 12 methyl carbons and among these carbons 10 were attributed to amino acid residues and therefore, the remaining 2 methyl carbons should be present in the aliphatic chain. One methyl carbon resonated at δ_C_ 14.6 was assigned as a terminal methyl and another methyl carbon signal resonated at δ_C_ 23.1 formed side chain with the fatty acid. The position of the later methyl group was located at C-11 of the fatty acid unit by the analysis of TOCSY and HMBC correlations. Based on the composition of the peptide moiety and the molecular weight of the molecule, the fatty acid chain was primarily determined to be a C_14_ fatty acid amidated to the *N*-terminal amine of the peptide. The chain length of the fatty acid was further confirmed from the acid hydrolysis of **1** followed by APCI-MS analysis (*m/z* at 243.02 [M − H]^−^) (SI). Thus, the 3-β-hydroxyl fatty acid was found as 3-β-hydroxy-11-methyltridecanoic acid in **1 **with an optical rotation value 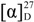 −29 (0.1, MeOH) (SI). The lipopeptide structure of **1** was then corroborated constructing the sequence of Leu-Leu-Asp-Val-Leu-Leu-Glu-fatty acid by two dimensional ROESY and HMBC experiments which showed long range proton-proton and proton-carbon correlations and correlations between amide protons and α-protons. 

Gageostatin B (**2**) was isolated as an amorphous solid and the molecular formula was determined to be C_53_H_95_N_7_O_14_ based on the high-resolution ESIMS peak at *m/z* 1076.6831 [M + Na]^+^. Preliminary, NMR analysis showed a close similarity between the spectra of compounds **1** and **2**, indicating a lipopeptide nature of **2**. However, a methyl and a methine carbons resonated at δ_C_ 11.9 and 37.8 were observed in the ^13^C NMR spectra of **2**, which were not observed in compound **1**. These carbons could be located either on the alkyl chain or on the peptide chain. Therefore, a detailed investigation on the structure of compound **2** was undertaken by running a series of 1D and 2D NMR experiments. The detailed NMR data analysis pointed to the same amino acids sequence between the two compounds. Therefore, these additional methyl and methine groups must be located in the aliphatic chain. The position of the methyl group was then corroborated at C-9 of the fatty acid unit by the analysis of TOCSY and HMBC correlations. Furthermore, the LC-MS analysis (*m/z* at 257.08 [M − H]^−^) of the hexane phase of the acid hydrolysate of **2**, confirmed the chain length of the fatty acid in compound **2**. These extensive NMR data along with LC-MS results confirmed the presence of 3-β-hydroxy-9,11-dimethyltridecanoic acid in **2 **with an optical rotation value 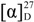 −6 (0.25, MeOH). The search result from the natural products database system suggested this fatty acid is a new fatty acid derivative. Finally, the complete chemical structure of compound **2** was constructed by the detailed analysis of COSY, TOCSY, HMBC and ROESY correlations (Supplementary Information). 

Gageostatin **3** was isolated as an amorphous solid and the molecular formula of C_51_H_89_N_7_O_13_ was deduced based on high-resolution ESIMS measurement at *m/z* 1030.6423 [M + Na]^+^. The detailed ^1^H and ^13^C data analyses were highly indicative of the lipopeptide nature of **3 **and found to possess same amino acids as **2**. However, the existence of signals at δ_H_ 6.01 (d, *J* = 15.5) and 6.79 (dt, *J* = 15.5, 8.5) in the ^1^H NMR data, which showed HSQC cross peaks with carbons resonated at δ_C_ 124.7 and 146.5, respectively, suggested the presence of a conjugated double bond in **3**. As the amino acids sequence was found to be same as **2**, corroborated by the TOCSY, HMBC and ROESY correlations, the double bond must be located at the fatty acid moiety. The structure of the fatty acid was then established with the help of TOCSY and HMBC correlations. The olefinic protons at δ_H_ 6.01 and 6.79 showed HMBC correlations to the carbonyl carbon resonated at δ_C_ 169.1. Furthermore, the olefinic proton at δ_H_ 6.01 showed TOCSY and HMBC correlations with the later part of fatty acid chain. These correlations were sufficient to locate the position of the conjugated diene C-2/C-3 position of the fatty acid moiety in **3**. The large coupling constant value of this conjugated diene suggested *E* configuration. Thus, the unsaturated fatty acid was assigned as (*E*)-7,9-dimethylundec-2-enoic acid in compound **3**. This fatty acid was also found as a new derivative, which has never been described before from the natural sources. The fatty acid moiety was then connected with the peptide sequence at the glutamic acid side with the help of HMBC correlations.

**Figure 3:**
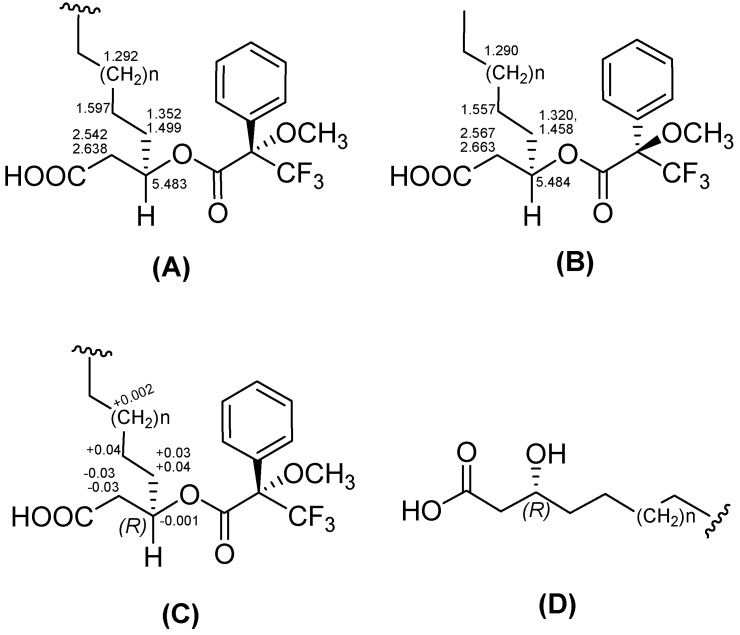
Absolute stereochemistry determination of 3-hydroxy fatty acid in **1**. (**A**) ^1^H NMR data of *S*-MTPA and (**B**) *R*-MTPA esters; (**C**) Δδ_H_ (δ*_S_* − δ*_R_*) values of derivatized products; (**D**) *R*-configuration of C-3 of fatty acid in **1**.

The absolute configuration of the amino acid residues in **1**–**3 **was determined by acid hydrolysis and Marfey’s method (SI) [21]. This method unambiguously established the configuration of each of Leu, Asp, and Glu to be l-form and Val to be d-form. In addition, the absolute configuration of **1** at selected stereocenter at C-3 of fatty acid moiety was determined by acid hydrolysis, followed by Mosher’s MTPA method [22] and assigned based on proton resonances of the *S*–MTPA and *R*–MTPA ester derivatives. A consistent distribution of positive and negative Δδ_H_ values (Δδ_H_ = δ*_S_* − δ*_R_*) around C-3 allowed the assignment of *R*-configuration for C-3 position (Figure 3) [23]. This result was also supported by the literature reviews, as (*R*)-3-hydroxy fatty acid displays negative molecular rotation in MeOH [24,25]. The absolute stereochemistry at 3-hydroxy position of the fatty acid in **2** was determined to possess *R* configuration by the comparison of optical rotation value with that of fatty acid in **1 **(Supplementary Information).

### 2.3. Antimicrobial Activities

Minimum growth inhibitory activity of compounds **1**–**3 **and **1**+**2 **was evaluated against both Gram positive and Gram negative bacteria and fungi following broth dilution assay [26]. Different growth conditions of bacteria and fungi were maintained while culturing these microorganisms [27,28]. It was found that mixed compounds **1 **+ **2** displayed better activity than individuals (Table 2). Moreover, compound **3** possessing an unsaturated fatty acid displayed less activity than **1**, **2** and **1 **+ **2**. It is noteworthy that these compounds **1**–**3 **and **1 **+ **2** showed better activity against fungi compared to bacteria.

**Table 2:** Minimum Inhibitory Concentrations (MICs) of **1**–**3**.

	MICs (µg/mL)				
Microorganisms	1	2	1 + 2	3	P.C.
**Fungi**					
*R. solani*	4	8	4	32	1
*C. acutatum*	8	8	4	16	1
*B. cinera*	4	8	4	32	1
**Gram Positive Bacteria**					
*S. aureus*	16	16	8	64	2
*B. subtilis*	16	32	16	32	2
**Gram Negative Bacteria**					
*S. Typhi*	16	32	32	32	2
*P. aeruginosa*	16	16	8	64	2

P.C.: Positive control (Azithromycin for Bacteria and Amphotericin B for Fungi).

### 2.4. Cytotoxic Properties

The cytotoxicity of compounds **1**–**3** and mixed compounds **1 **+ **2** was also evaluated against a panel of six human cancer cell lines (breast cancer: MDA-MB-231, colon cancer: HCT15, prostate cancer: PC-3, lung cancer: NCI-H23, stomach cancer: NUGC-3, and renal cancer:ACHN) according to a sulforhodamine B (SBR) assay [29] and found to display moderate cytotoxicity (Table 3). Compounds **1**–**3** showed similar activity against all cell lines but mixed compounds **1 **+ **2** showed better inhibitory activity. In particular, mixed compounds **1 **+ **2** displayed significant activity against lung cancer (NCI-H23) with a GI_50_ value of 4.6 µg/mL.

**Table 3:** Human Cancer Cell Line Inhibition Values (GI_50_) of **1**–**3**.

Cancer Cell Lines	(GI_50_, µg/mL)				
	1	2	1 + 2	3	ADR ^a^
Breast cancer: MDA-MB-231	14.9	16.1	10.5	11.2	0.56
Colon cancer: HCT-15	11.4	18.3	10.9	23.2	0.33
Prostate cancer: PC-3	10.8	19.4	12.0	11.7	0.91
Lung cancer: NCI-H23	11.2	11.7	4.6	10.9	0.71
Stomach cancer: NUGC-3	11.8	13.9	10.1	10.5	0.53
Renal cancer: ACHN	11.5	18.4	10.7	12.3	0.51

^a^ ADR means adriamycin as standard.

## 3. Experimental Section

### 3.1. General Experimental Procedures

UV spectra were obtained on a Shimadzu UV-1650PC spectrophotometer. IR spectra were recorded on a JASCO FT/IR-4100 spectrophotometer. Optical rotations were measured on a JASCO (DIP-1000) digital polarimeter. Nuclear magnetic resonance spectroscopic data were acquired on a Varian Unity 500 spectrometer. High-resolution ESIMS were recorded on a hybrid ion-trap time-of-flight mass spectrometer (Shimadzu LC/MS-IT-TOF). HPLC was conducted with a PrimeLine Binary pump with RI-101(Shodex) and Variable UV Detector (M 525). Semi-preparative HPLC was performed using ODS (YMC-Pack-ODS-A, 250 × 10 mm i.d, 5 µm) and silica (YMC-Pack-SIL, 250 × 10 mm i.d, 5 µm) columns. Analytical HPLC was conducted on an ODS column (YMC-Pack-ODS-A, 250 × 4.6 mm i.d, 5 µm). All solvents used were either spectral grade or distilled prior to use. Natural sea water was collected from East Sea of South Korea at depths of 20 m. 

### 3.2. Isolation and Identification of the Strain 109GGC020

The strain 109GGC020 was isolated from a sediment sample collected from Gageocho, in the Republic of Korea, in 2010, by serial dilution techniques and grown on Bennett’s media agar plate (For 1 L 100% natural sea water media composition was 1% dextrose, 0.1% yeast extract, 0.1% beef extract, 0.2% tryptone, 1.8% agar and pH adjusted to 7.1). The strain was then identified as *Bacillus subtilis* on the basis of 16S rRNA sequence analysis. The sequence was deposited in GenBank under accession number JQ927413. This strain is currently preserved in the Microbial Culture Collection, KIOST, with the name *Bacillus subtilis* 109GGC020 under the curatorship of Hee Jae Shin.

### 3.3. Seed and Mass Cultures of the Strain

The seed culture of the strain 109GGC020 was performed in triplicate into 100 mL flasks, containing 50 mL medium. An aliquot (0.2% v/v) from the seed culture was inoculated aseptically into 2 L flasks (total 50 flasks) containing 1.2 L sterilized culture medium (same composition as above). The production culture was incubated at 28 °C for 7 days.

### 3.4. Extraction and Isolation of Compounds

The cells were separated from the culture broth by centrifugation and the broth was extracted with EtOAc (2 times). The EtOAc layer was evaporated to dryness under reduced pressure at 40 °C. The obtained crude extract (11.8 g) was subjected to an ODS open column chromatography followed by stepwise gradient elution with MeOH–H_2_O (v/v) (1:4, 2:3, 3:2, 4:1 and 100:0) as eluent. The 100% MeOH fraction was again subjected to further fractionations and purification by C_18_ semi-preparative and analytical HPLC using solvent system 95% MeOH in H_2_O to yield pure compounds **1**–**3**.

Gageostatin A (**1**)**: **HPLC retention time (42 min); amorphous solid; 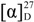 +52 (*c* 0.1, MeOH); IR (MeOH) ν_max_ 3291 cm^−1^ (NH) and 1646–1737 cm^−1^ (CO), 2930 cm^−1^; ^1^H and ^13^C NMR data (CD_3_OD), Table 1; HRESIMS *m/z* 1062.6691 [M + Na]^+^.

Gageostatin B (**2**)**: **HPLC retention time (44 min); amorphous solid; 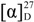 +53 (*c* 0.1, MeOH); IR (MeOH) ν_max_ 3293 (br), 2927, 1742 cm^−1^; ^1^H and ^13^C NMR data (CD_3_OD), Table 1; HRESIMS *m/z* 1076.6831 [M + Na]^+^.

Gageostatin C (**3**)**: **HPLC retention time (50 min); amorphous solid; 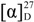 +16 (*c* 0.1, MeOH); IR (MeOH) ν_max_ 3373 (br), 2970, 1660 cm^−1^; ^1^H and ^13^C NMR data (CD_3_OD), Table 1; HRESIMS *m/z* 1030.6423 [M + Na]^+^.

### 3.5. Acid Hydrolysis of Compounds **1**–**3**

Compound **1** (2.1 mg) was dissolved in 6N HCl (0.50 mL) and stirred at 120 °C for 23 h. After cooling, the solution was diluted with water, and the products were extracted with chloroform. The chloroform extract was concentrated under a stream of N_2_ and aqueous part was evaporated to dryness under reduced pressure. The chloroform extract containing fatty acid was derivatized with Mosher’s reagent to determine absolute configuration at the stereocenter C-3 of 3-hydroxy fatty acid and the aqueous part was subjected for amino acids analysis by Marfey’s method. In the similar manner, compounds **2** (0.8 mg) and **3** (0.2 mg) were hydrolyzed and analyzed.

Compound **1a **(1.5 mg): colorless oil; ^1^H NMR data (CD_3_OD) δ_H_ 2.35 (H-2a, dd, *J* = 15.0, 8.5), 2.44 (H-2b, dd, *J* = 15.5, 5.0), 3.97 (H-3, m), 1.34 (H-4a, m, overlapped), 1.45 (H-4b, m, overlapped), 1.47 (H_2_-5, m, overlapped), 1.29 (H_2_-6-H_2_-9, brds.), 1.17 (H-10, m), 1.51 (H-11, m, overlapped), 1.29 (H-12, m, overlapped), 0.86 (H_3_-13, t, *J* = 5.5), 0.87 (H_3_-14, d, *J* = 6.5); APCI-MS *m/z* 243.04 [M − H]^−^; molecular rotation 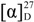 −29 (0.1, MeOH).

### 3.6. Preparation of the *(S)*- and *(R)*-MTPA Esters of 3-Hydroxy Fatty Acids (**1*b*** and **1*c***)

Compound **1a **(0.7 mg) was dissolved in 200 µL of pyridine into a 4 mL reaction vial and stirred at room temperature (rt) for 10 min. To the vial, 20 µL of (*R*)-(−)-MTPA-Cl was added to prepare the (*S*)-MTPA ester (**1b**) of **1a**, and the mixture was stirred at rt for 16 h. Completion of the reaction was monitored by LC/MS. The reaction mixture was dried *in vacuo*, re-dissolved in EtOAc, washed with H_2_O, and purified by an analytical HPLC using 90% MeOH in H_2_O as eluent to obtain **1b** (0.4 mg). In an entirely analogous way, (*R*)-MTPA ester (**1c**) of **1a** was prepared by using (*S*)-(+)-MTPA-Cl. Analysis of the Δδ_H_ (δ*_S_* − δ*_R_*) values obtained by ^1^H NMR spectrum for each couple of diastereomeric MTPA esters, according to the Mosher model pointed to an *R*-configuration at C-3 of the fatty acid.

### 3.7. Advanced Marfey’s Analysis of **1**–**3**

The hydrolysates of **1**–**3** were re-suspended in H_2_O (100 µL). A 0.1% 1-fluoro-2,4-dinitrophenyl-5-l-alaninamide solution in acetone (Marfey’s reagent, 20 µL) and 1N NaHCO_3_ (10 µL) were added to a portion of the hydrolysate, and the mixture was heated at 40 °C for 2 h. The solution was cooled to room temperature, neutralized with 2N HCl (5 µL), and evaporated to dryness. The residue was re-suspended in H_2_O (50 µL) and analyzed by reversed-phase HPLC (YMC-Pack ODS, 250 × 4.6 mm, 5 µm, flow rate of 0.5 mL/min) using a linear gradient of 40% CH_3_CN in H_2_O containing 0.5% TFA in 60 min at 28 °C. Similarly, standard amino acids (both l and d) were derivatized with Marfey’s reagent and analyzed. The derivatized Leu, Asp, Glu residues in the hydrolysate of **1 **were eluted at the same retention time as the derivatized standard respective l-amino acids but not that of d-Val (30.0 min). 

### 3.8. Antimicrobial Assays

The antimicrobial activity of compounds **1**–**3** and **1 **+ **2** was determined by using a standard broth dilution assay against Gram positive and Gram negative bacteria and fungi. Antibacterial and antifungal tests were performed in nutrient broth and yeast maltose broth, respectively. A serial double dilution of each compound was prepared in 96-microtiter plates over the range of 0.5–256 µg/mL. An overnight broth culture of each strain was prepared and final concentration of organisms in each culture was adjusted to 1.5 × 10^8^ cfu/mL by comparing the culture turbidity with the 0.5 McFarland Standard. Culture broth (30 µL) was added to each dilution of compounds **1**–**3** and **1** + **2** and the final volume of each well was adjusted to 200 µL using the respective culture medium, and the plates were incubated 24 h at 37 °C for bacteria and 48 h at 30 °C for yeast. The minimum inhibitory concentration (MIC) is the lowest concentration of a sample at which the microorganism did not demonstrate visible growth, as indicated by the presence of turbidity.

### 3.9. Cytotoxicity Test

Cancer cell growth inhibitory activity of compounds **1**–**3** and mixed compounds **1** + **2** was performed according to a sulforhodamine B (SBR) assay. In brief, 96-well plate was loaded with selected cell lines (MDA-MB-231, HCT-15, PC-3, NCI-H23, NUGC-3, and ACHN) and tested samples (30, 10, and 3 µg/mL) were added. After incubation for 48 h, anchorage-dependant cells are fixed with 50% (wt/vol) trichloroacetic acid and stained for 60 min. Access dye was washed with SRB solution (0.4% sulforhodamine B in 1% acetic acid). The protein-bound dye is dissolved in 10 mM Tris base solution and absorbance was measured at 510 nm using a microplate reader. GI_50_ values of compounds **1**–**3** and **1 **+ **2** were then calculated using graphed prism software.

## 4. Conclusions

In summary, we discovered three new linear LPs from the culture broth of *B. subtilis.* The detailed structural analysis of these compounds indicated the same amino acid residues and sequence to the reported surfactins A–D [30]. However, the major differences between compounds **1**–**3** and surfactins were found in their molecular mass and structures. Compounds **1**–**3** were obtained as a linear form with all l-Leu, whereas surfactins A–D are cyclic peptides with l- and d-Leu. Moreover, the search result in a natural products database confirmed the characterization of new fatty acid moieties in compounds **2** and **3**. Surfactins have been reported to display wide range of bioactivities including antibacterial, antifungal, and anticancer activities [31,32,33,34]. Recently, surfactin like cyclic LPs have also been discovered and reported to display strong and dose-dependent antifungal activity against the plant pathogenic fungus *Fusarium oxysporum* [35]. Together with the pharmaceutical applications, LPs are also used as biosurfactants such as emulsifier in the food and cosmetic industries and bioremediation and dispersion of oil spills due to their biodegradability, low toxicity and environmental compatibility properties [36,37]. In our study, the antimicrobial activity results of **1**–**3 **indicated their possibilities to be drug candidates too. In particular, these LPs or their modified form would be utilized as antifungal agents as well as biosurfactants. 

## Supplementary Files

Supplementary File 1 Supplementary Information (PDF, 434 KB)
