# Supplementary material for: Gageostatins A–C, Antimicrobial Linear Lipopeptides from a Marine Bacillus subtilis
Source: Mar Drugs. 2014 Jan 31;12(2):871–85. doi: 10.3390/md12020871 (PMC3944520; doi:10.3390/md12020871)

Supplementary Information

Figure S1. ^{1}H NMR spectrum of **1** in CD_3OD at 500 MHz.

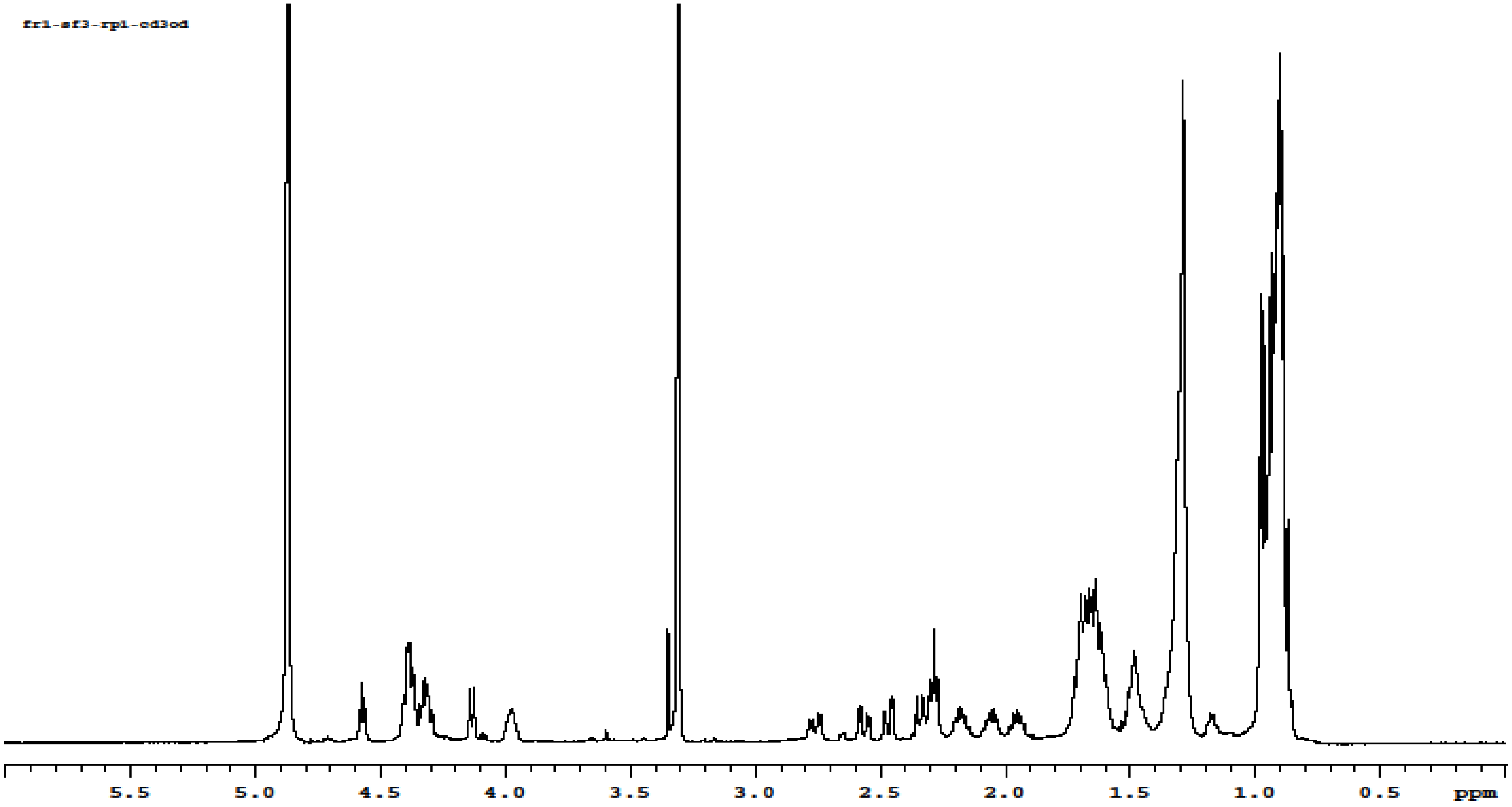

Figure S1. *Cont.*

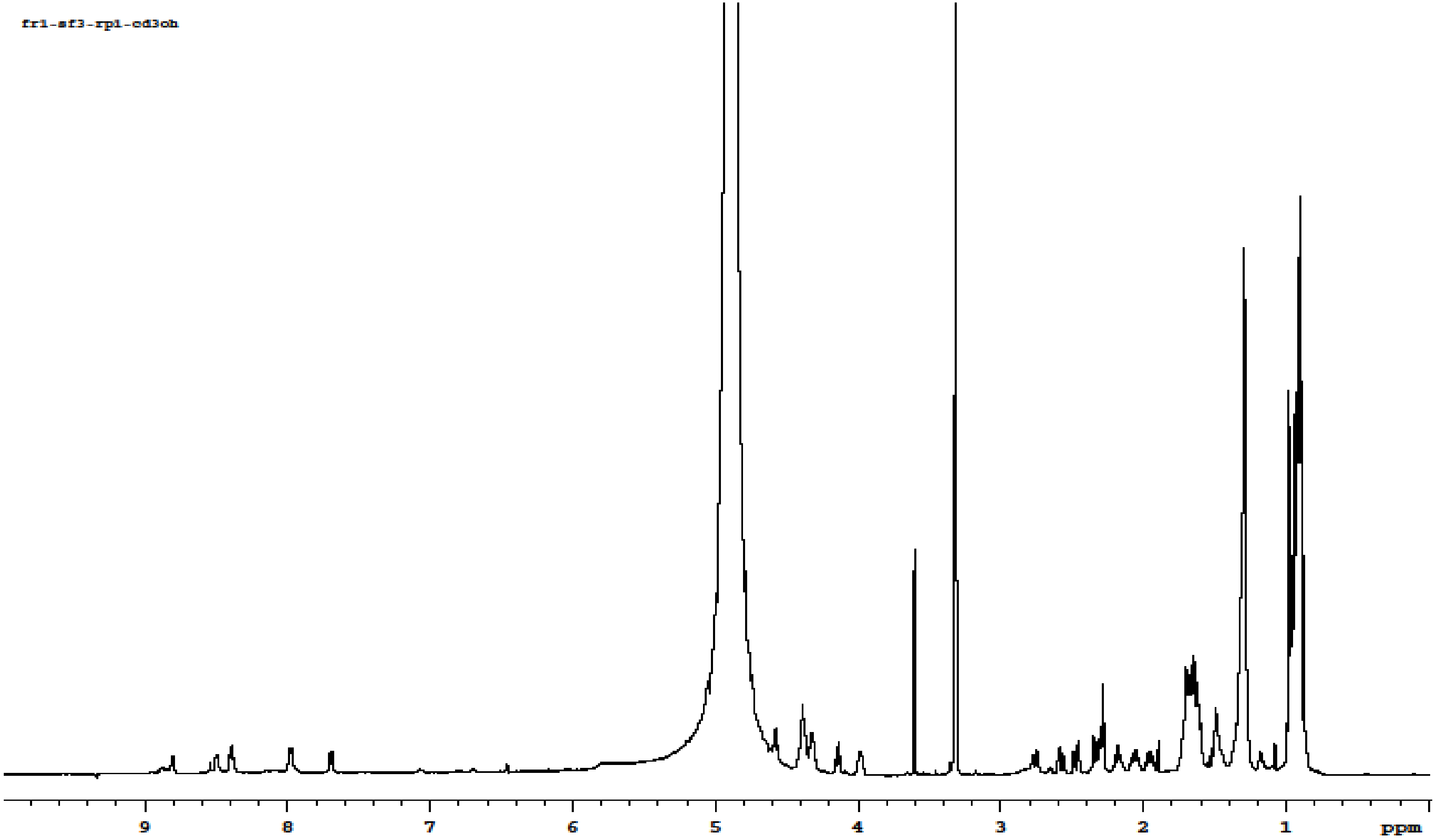

Figure S2. ^{13}C NMR spectrum of **1** in CD_3OD.

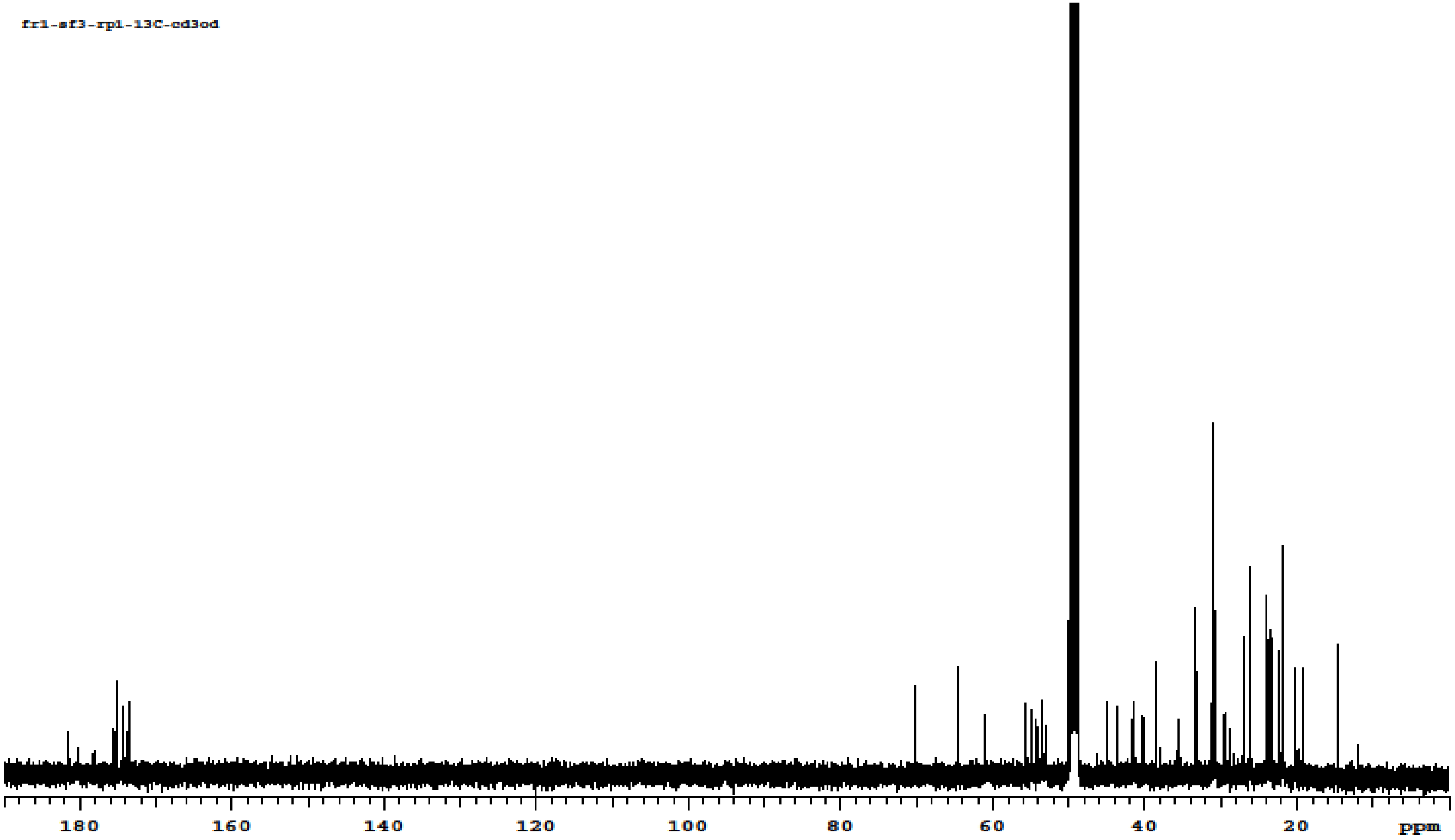

Figure S3. COSY spectrum of **1** in CD_3OD.

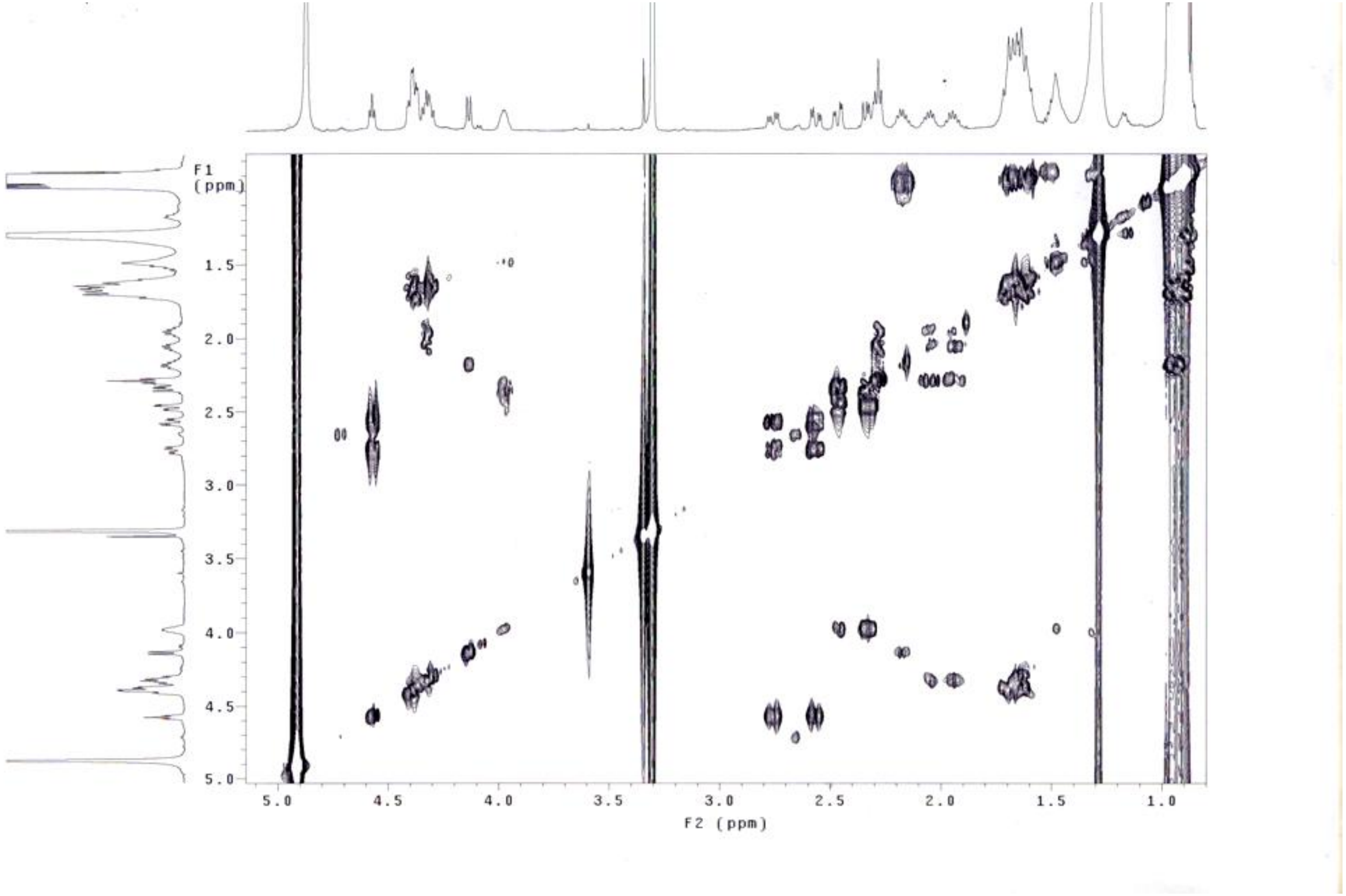

Figure S4. COSY spectrum of **1** in CD_3OH.

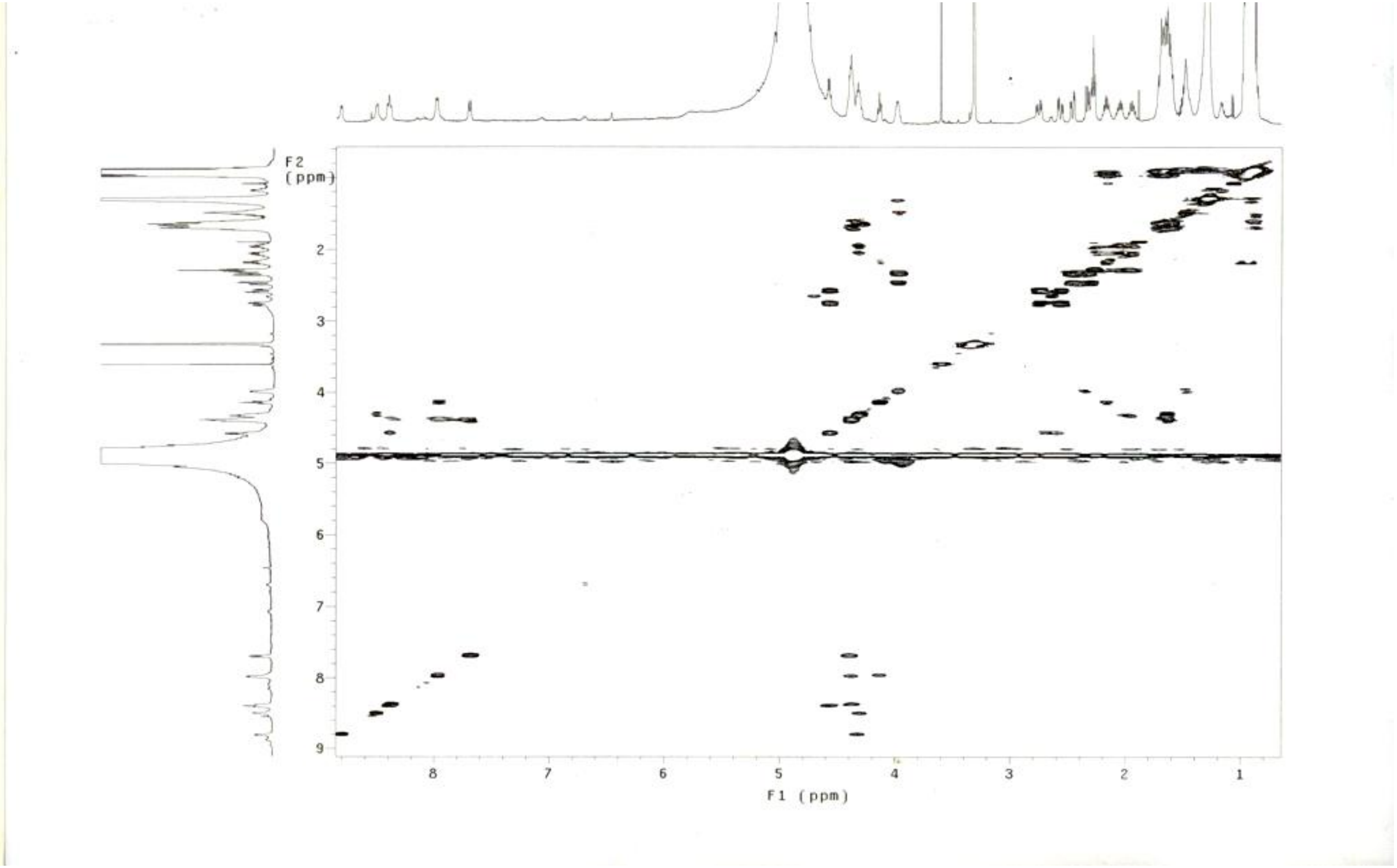

Figure S5. TOCSY spectrum of **1** in CD_3OH.

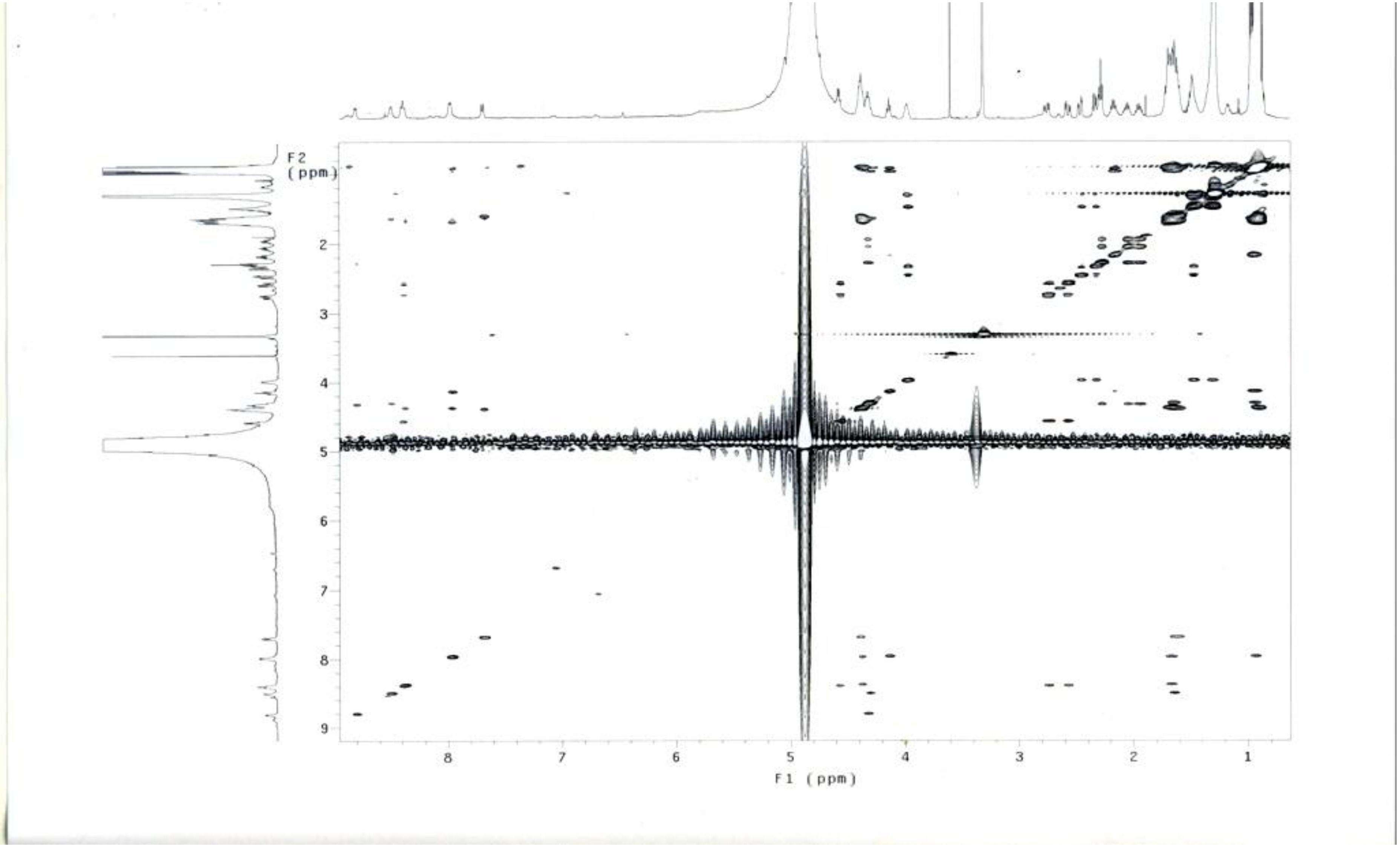

Figure S6. HSQC spectrum of **1** in CD_3OD.

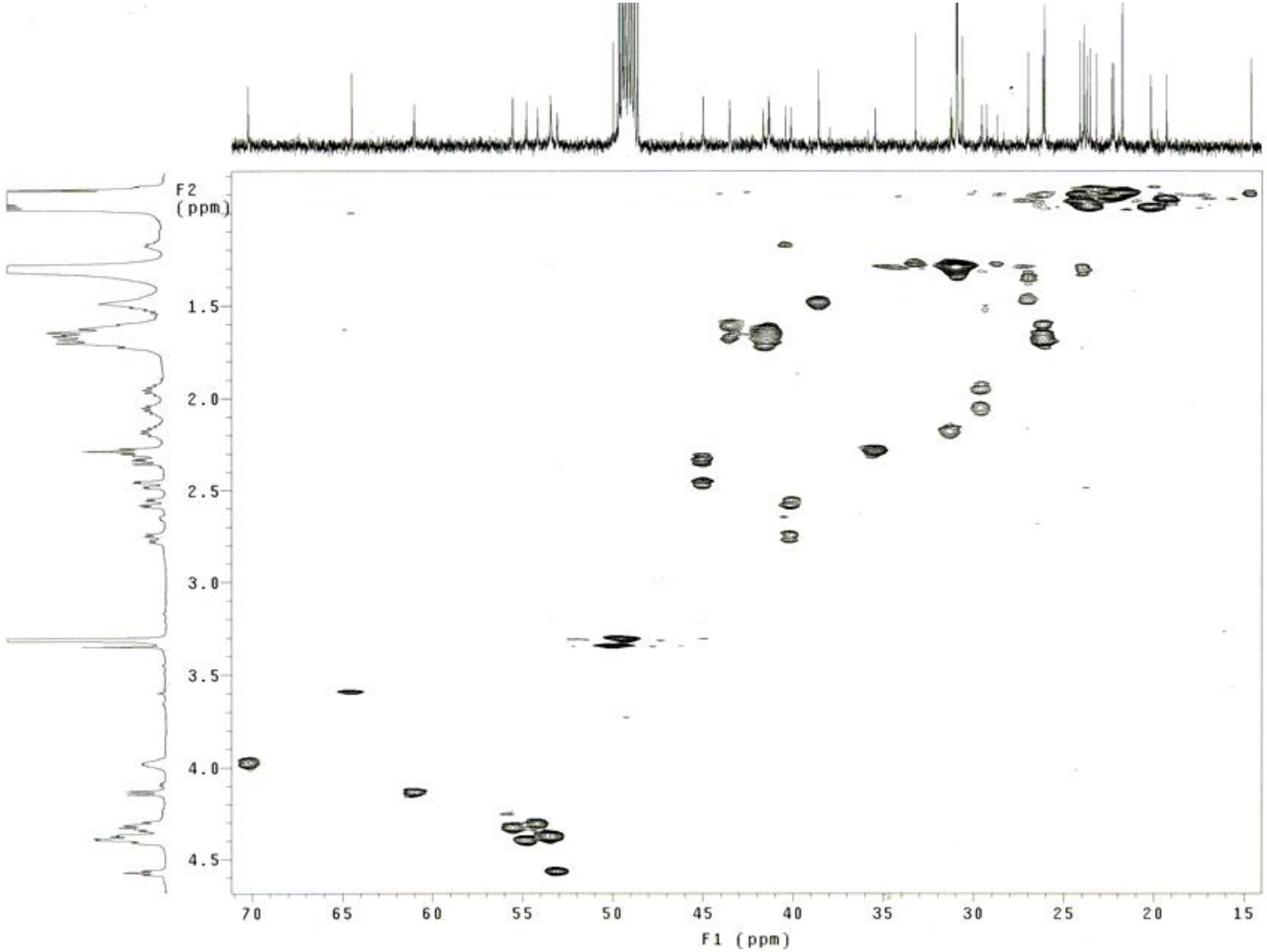

Figure S7. HMBC spectrum of **1** in CD_3OD.

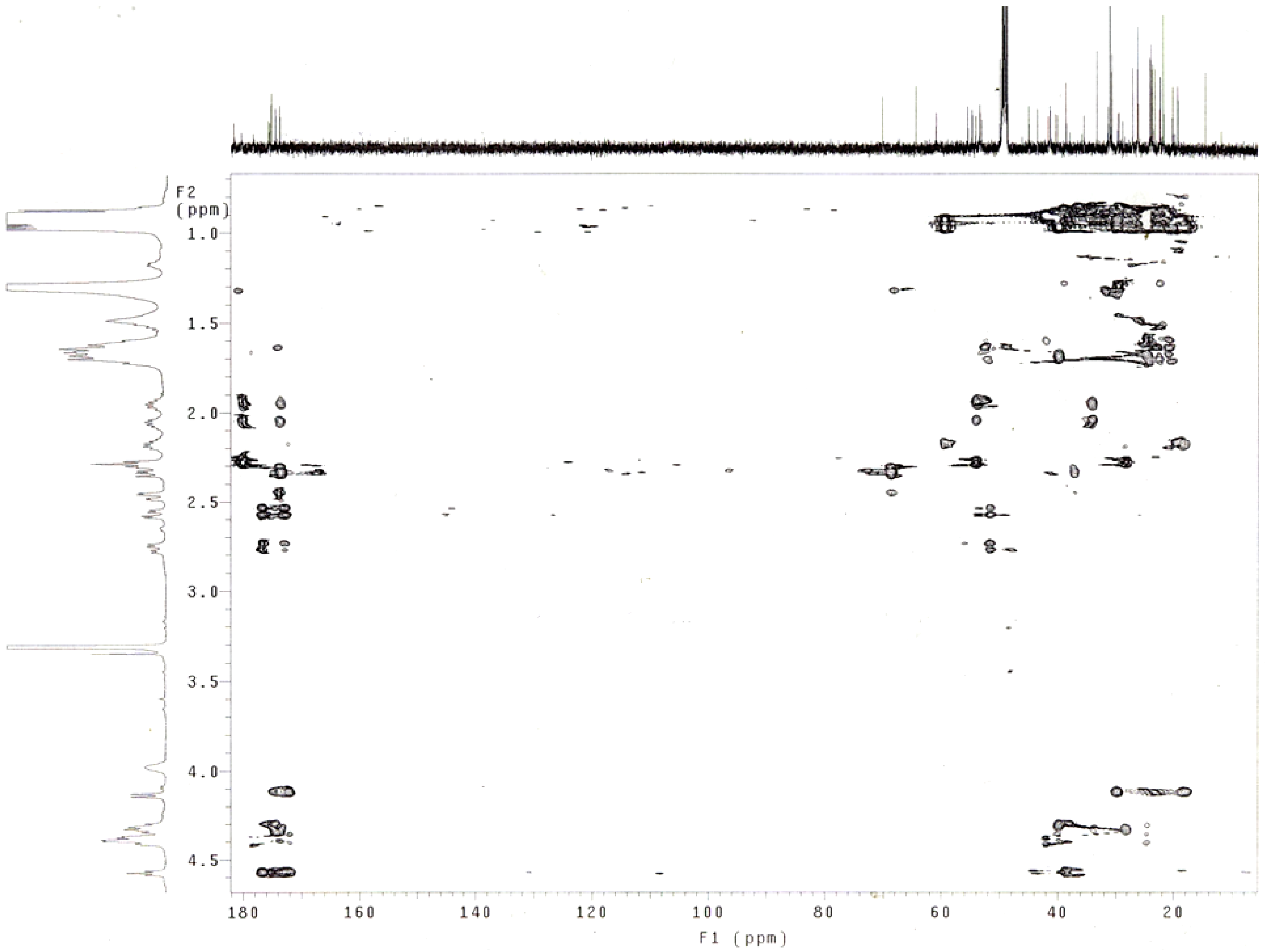

Figure S8. HMBC spectrum of **1** in CD_3OH.

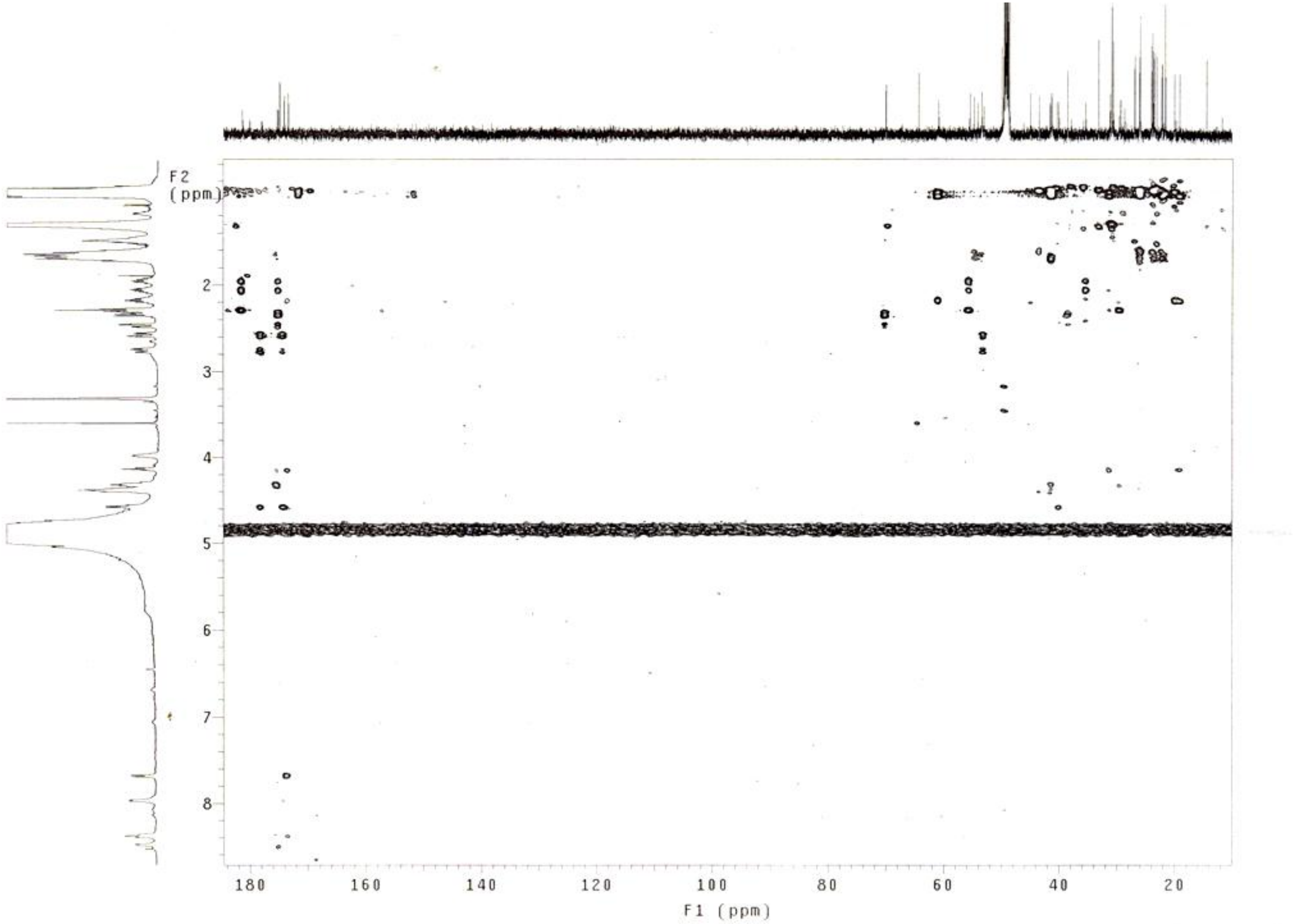

Figure S9. ^{1}H NMR spectrum of **2** in CD_3OD.

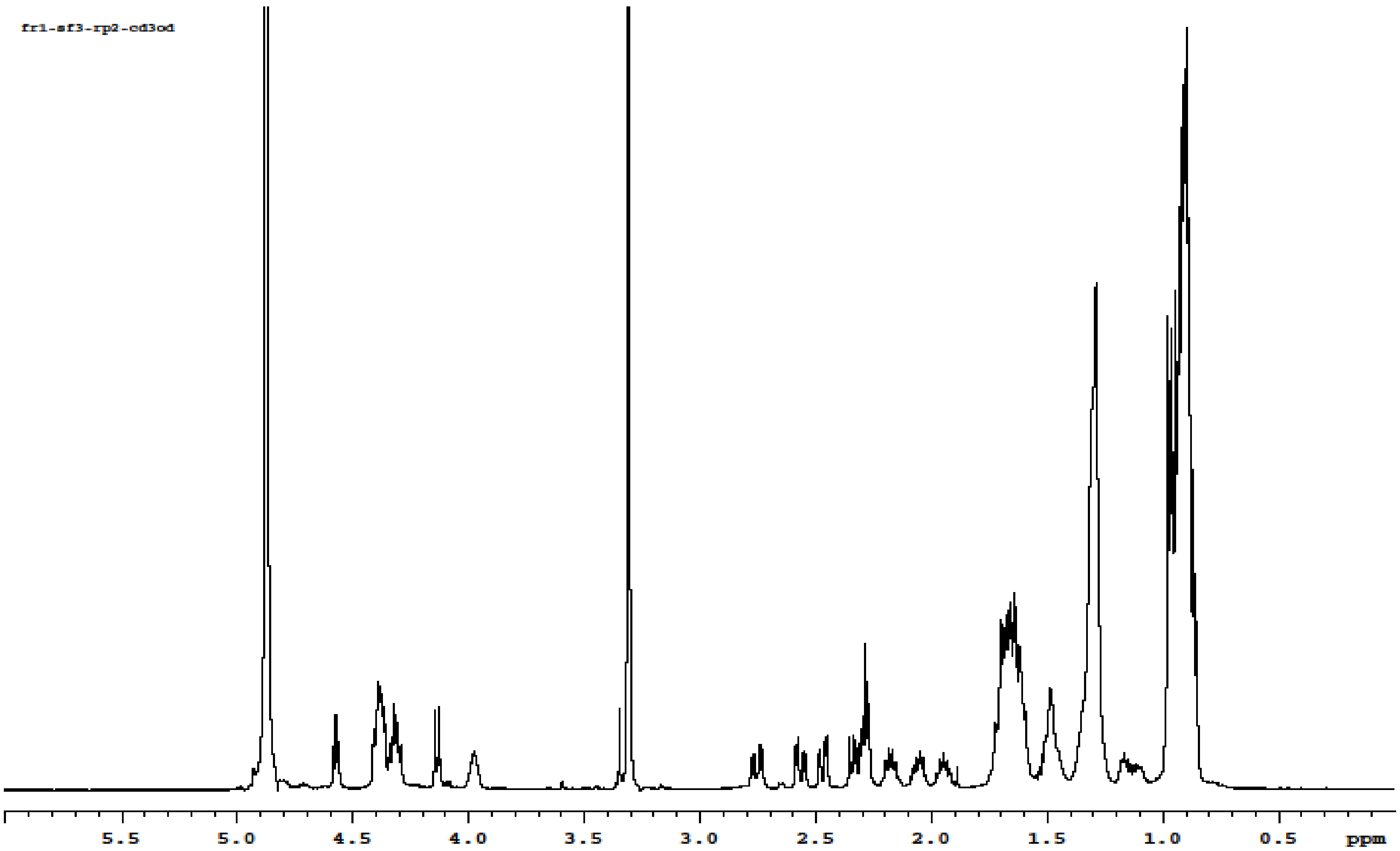

Figure S9. *Cont.*

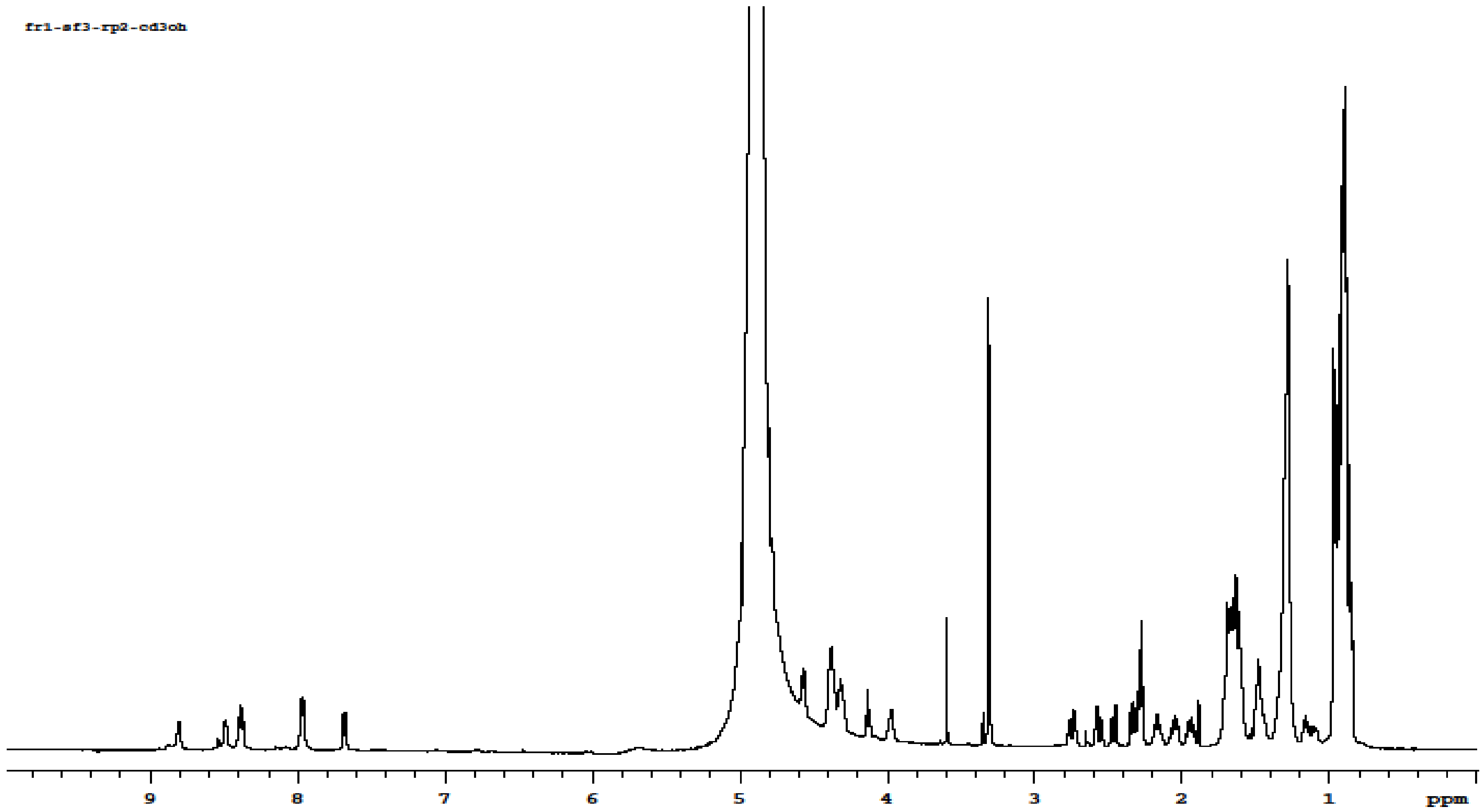

Figure S10. ^{13}C NMR spectrum of **2** in CD_3OD.

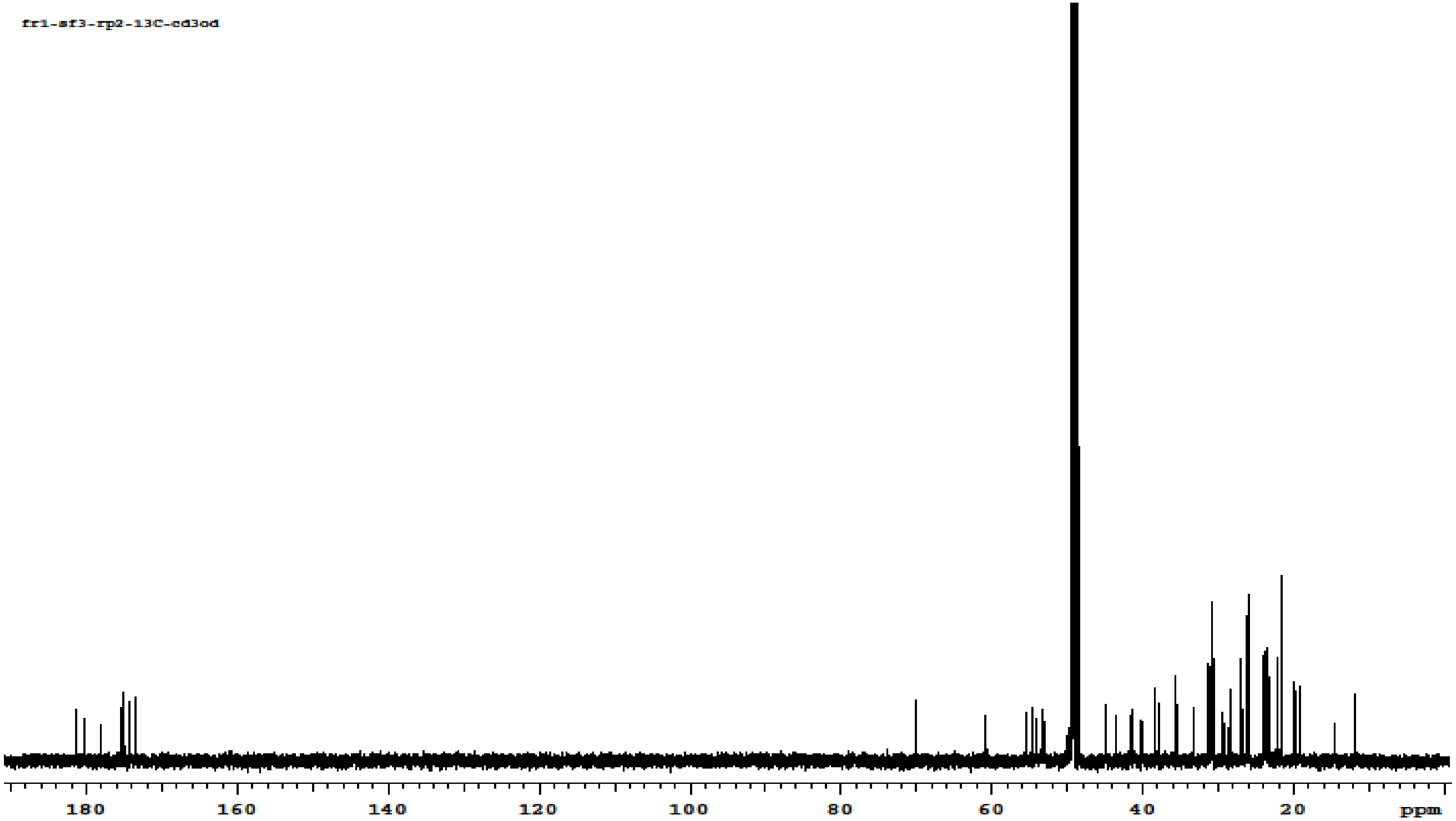

Figure S11. COSY spectrum of **2** in CD_3OD.

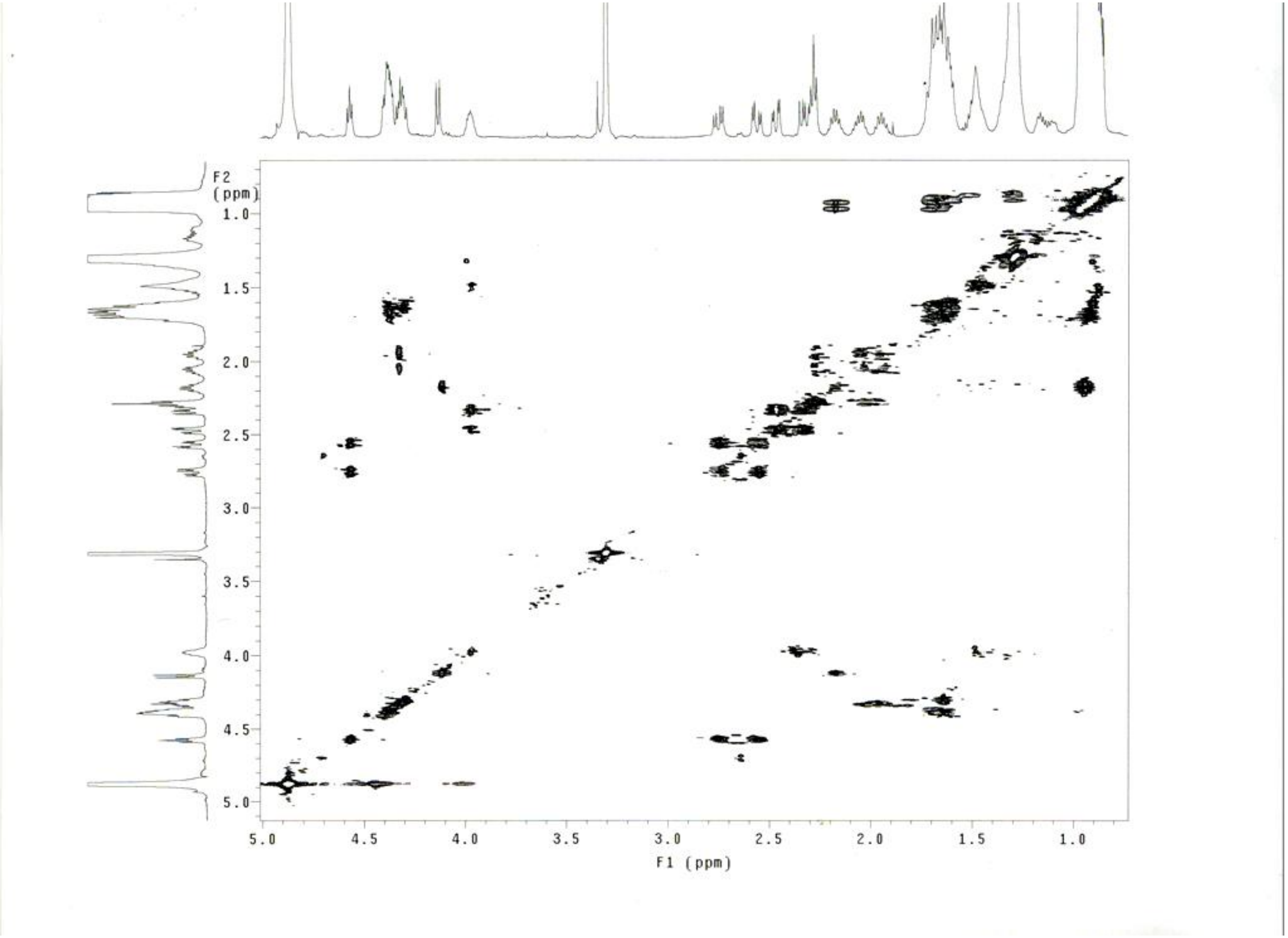

Figure S12. TOCSY spectrum of **2** in CD_3OH.

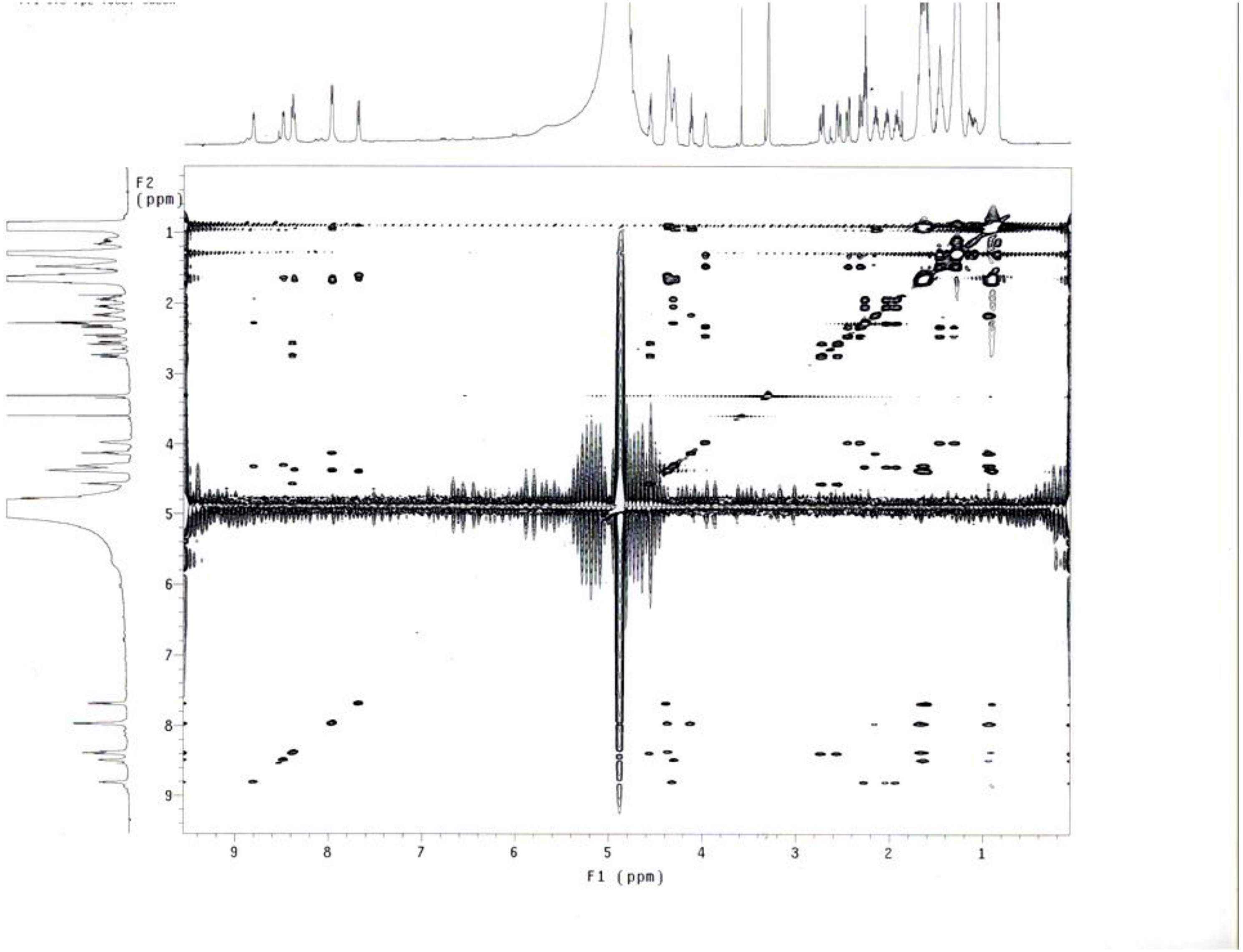

Figure S13. HSQC spectrum of **2** in CD_3OD.

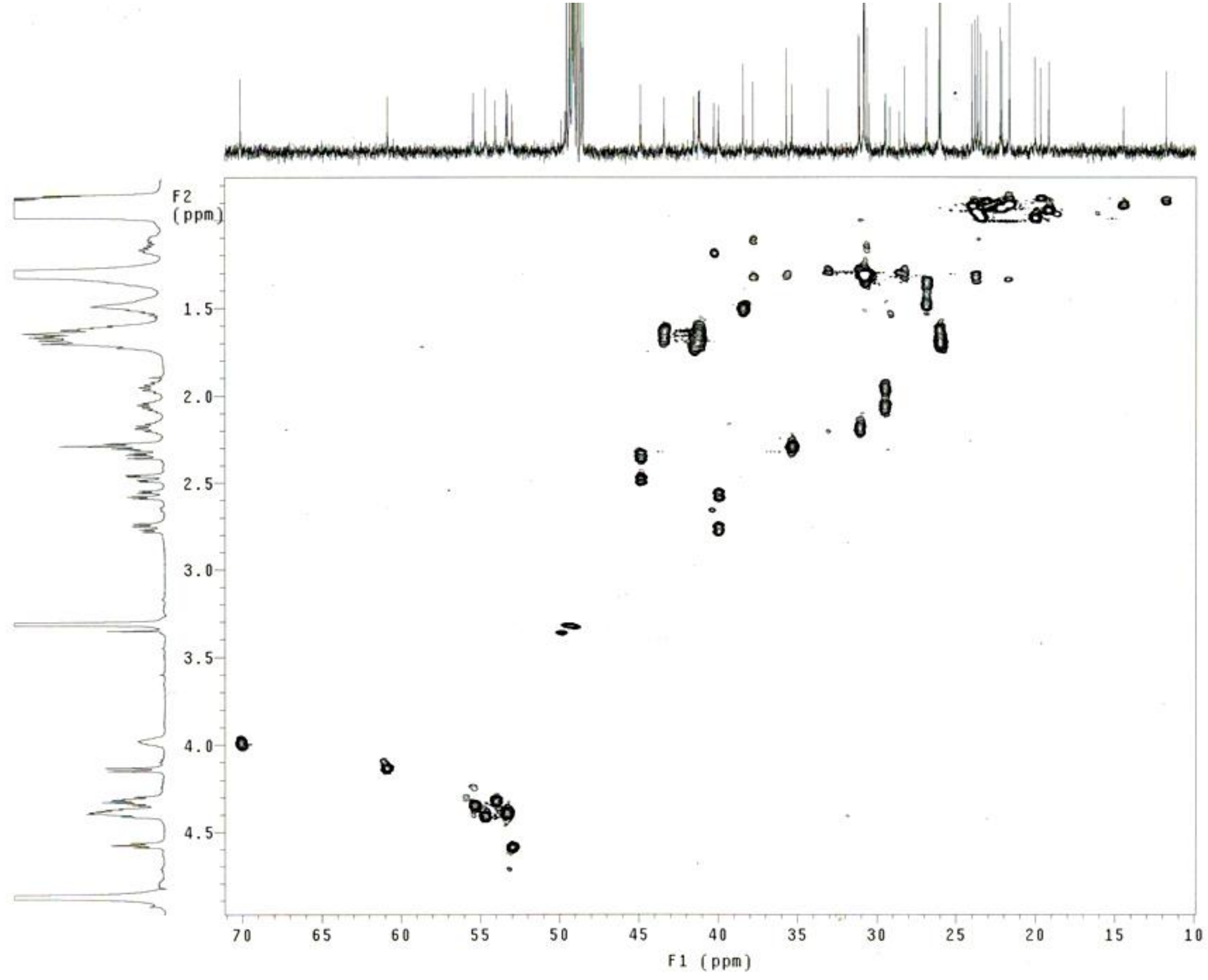

Figure S14. HMBC spectrum of **2** in CD_3OD.

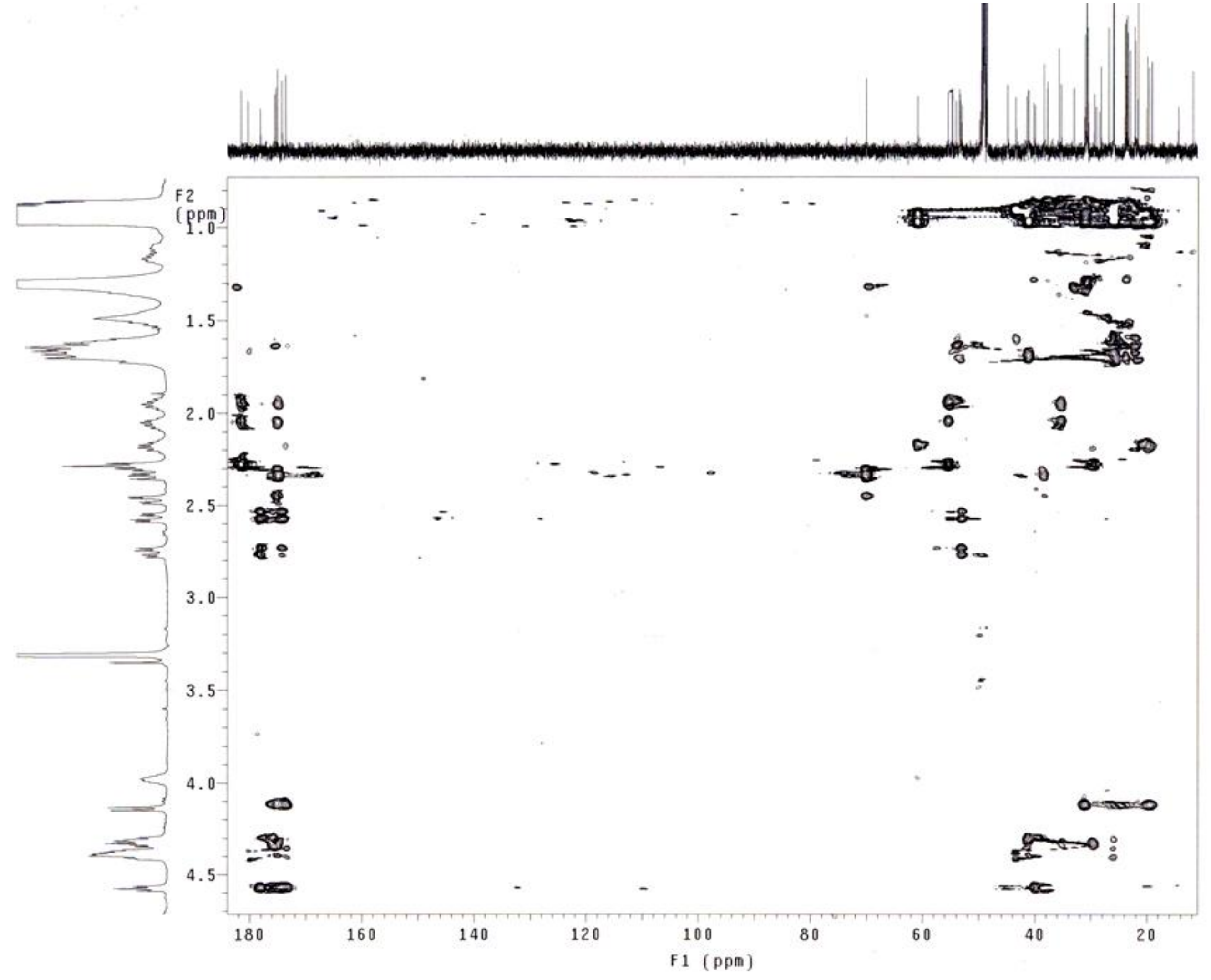

Figure S15. ROESY spectrum of **2** in CD_3OH.

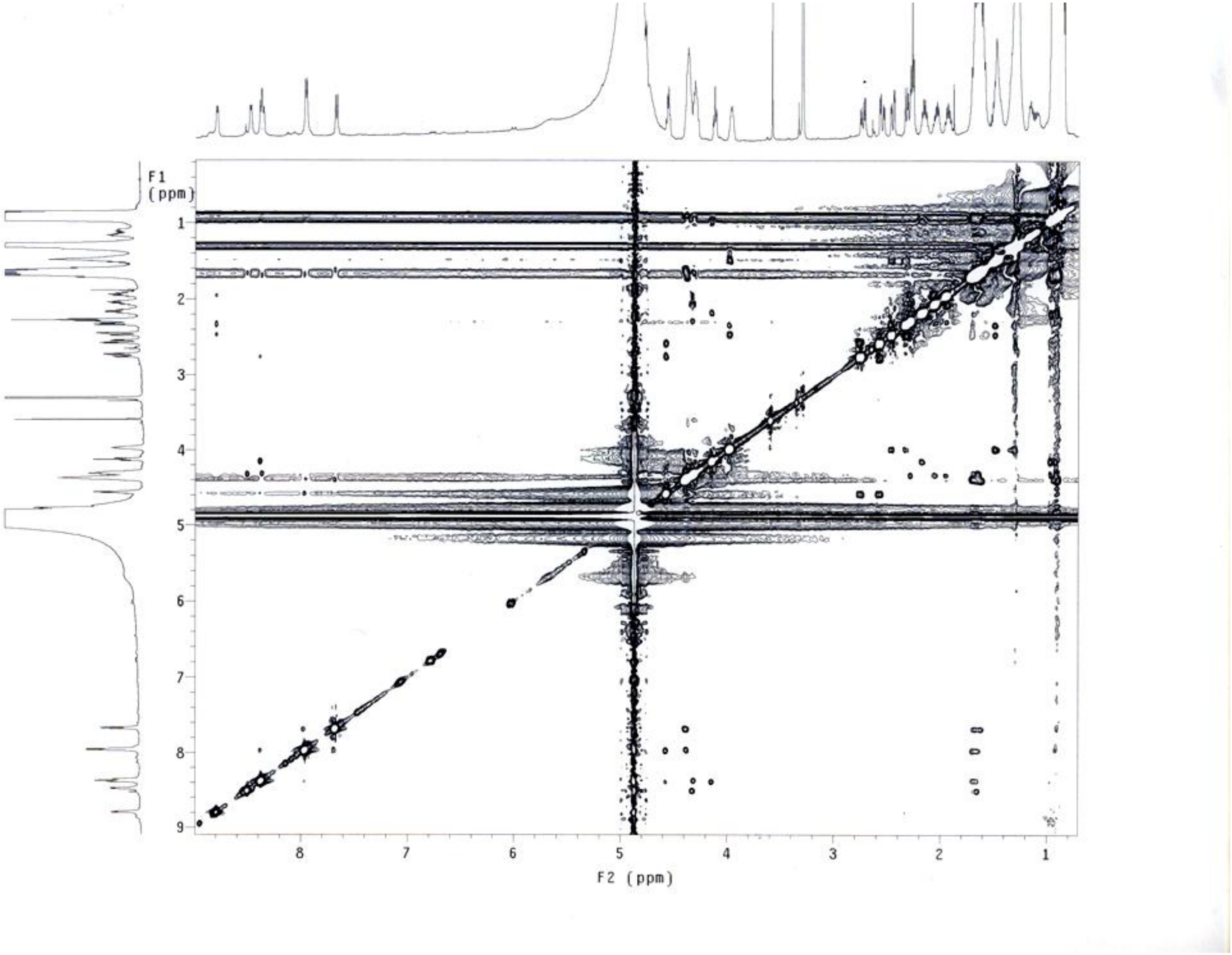

Figure S16. ^{1}H NMR spectrum of **3** in CD_3OD.

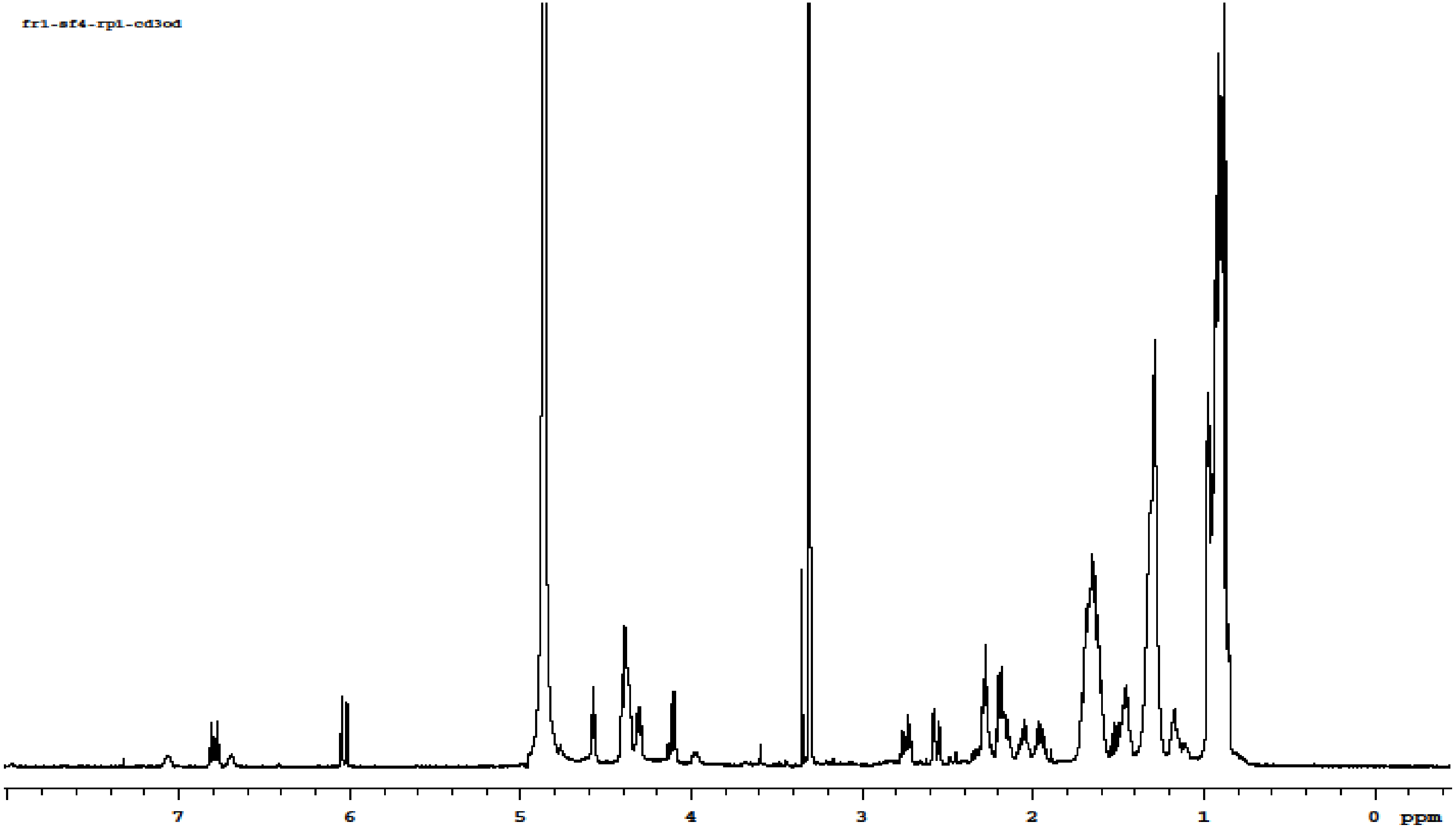

Figure S16. *Cont.*

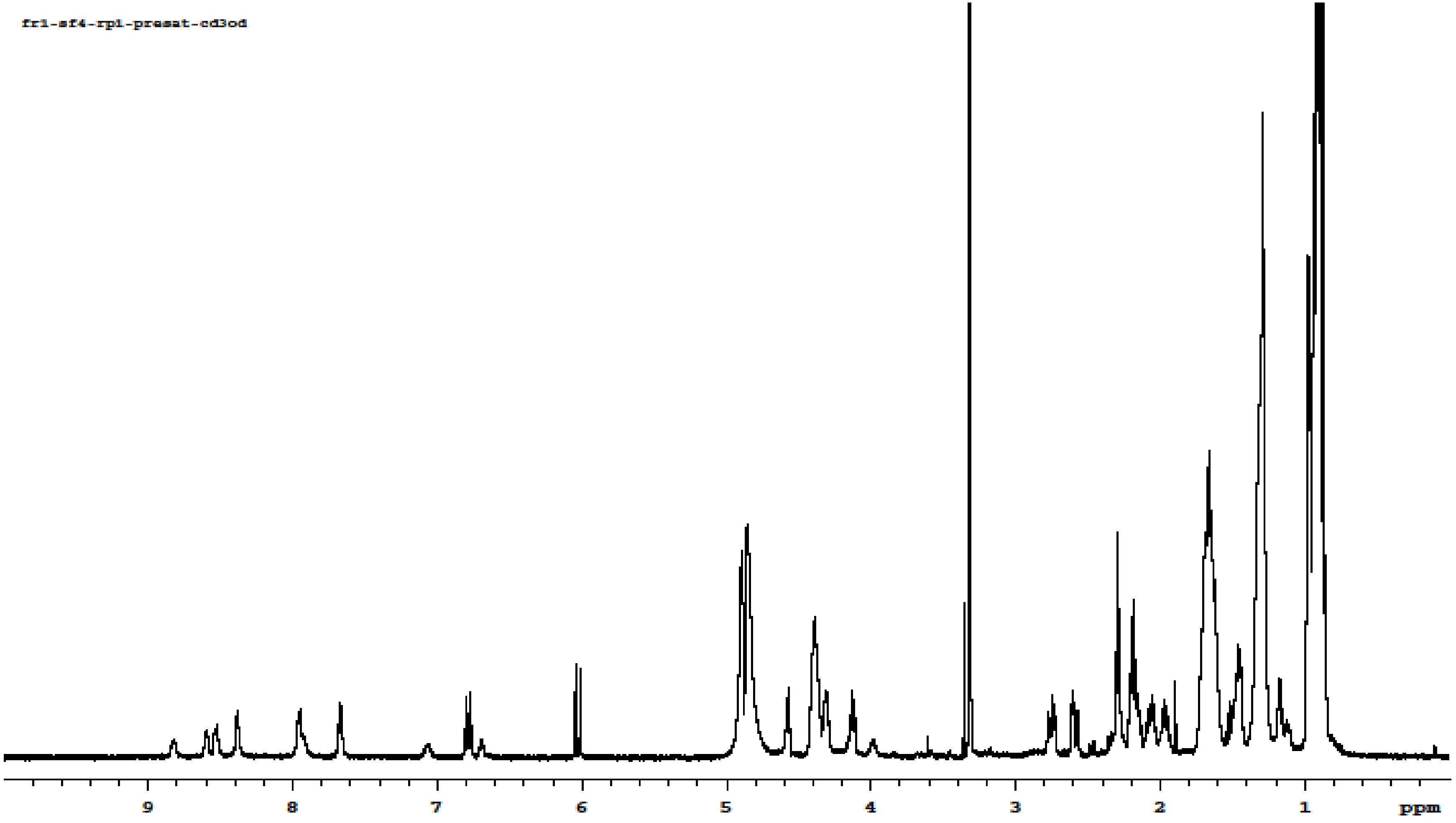

Figure S17. ^{13}C NMR spectrum of **3** in CD_3OD.

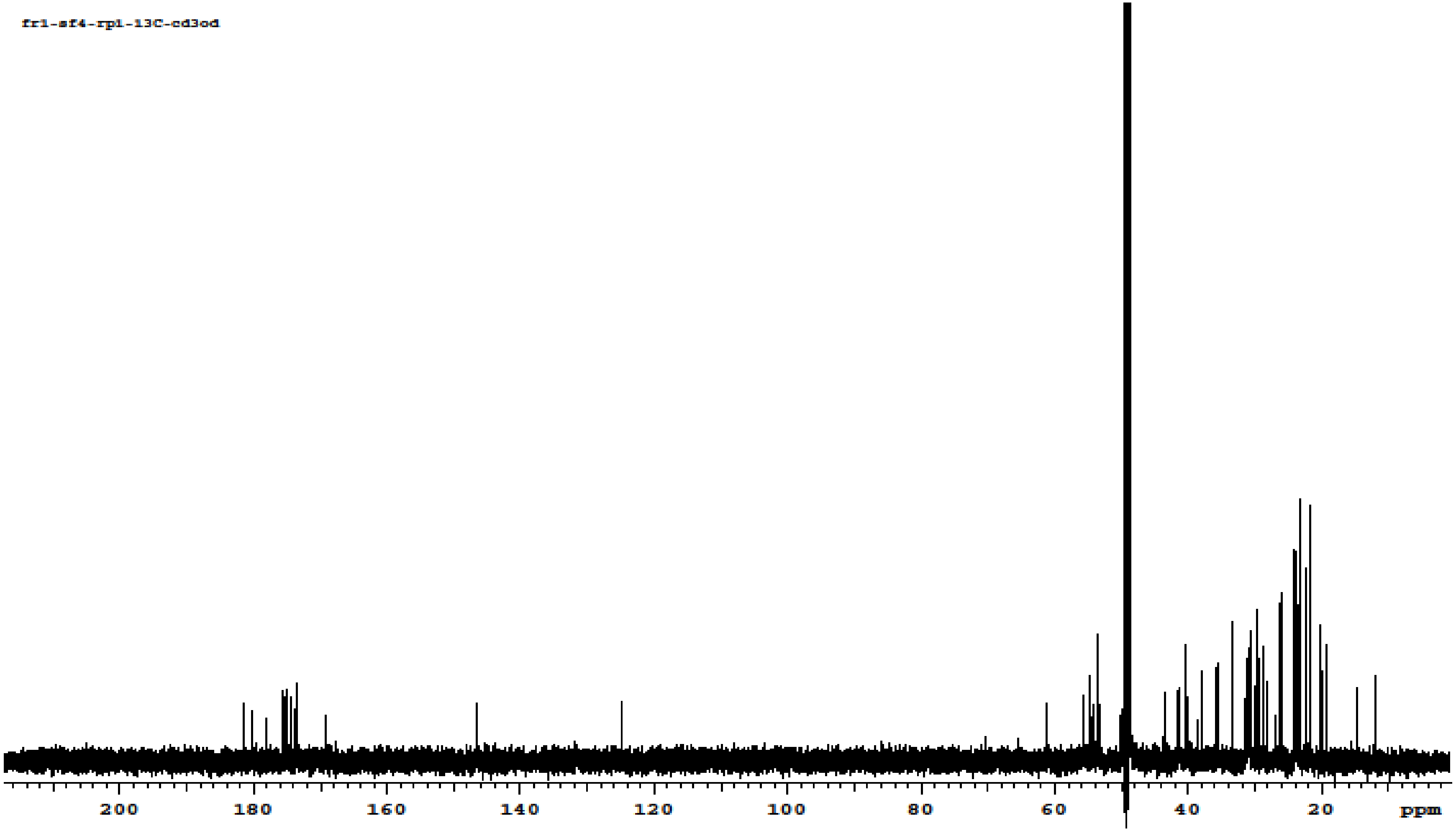

Figure S18. COSY spectrum of **3** in CD_3OD.

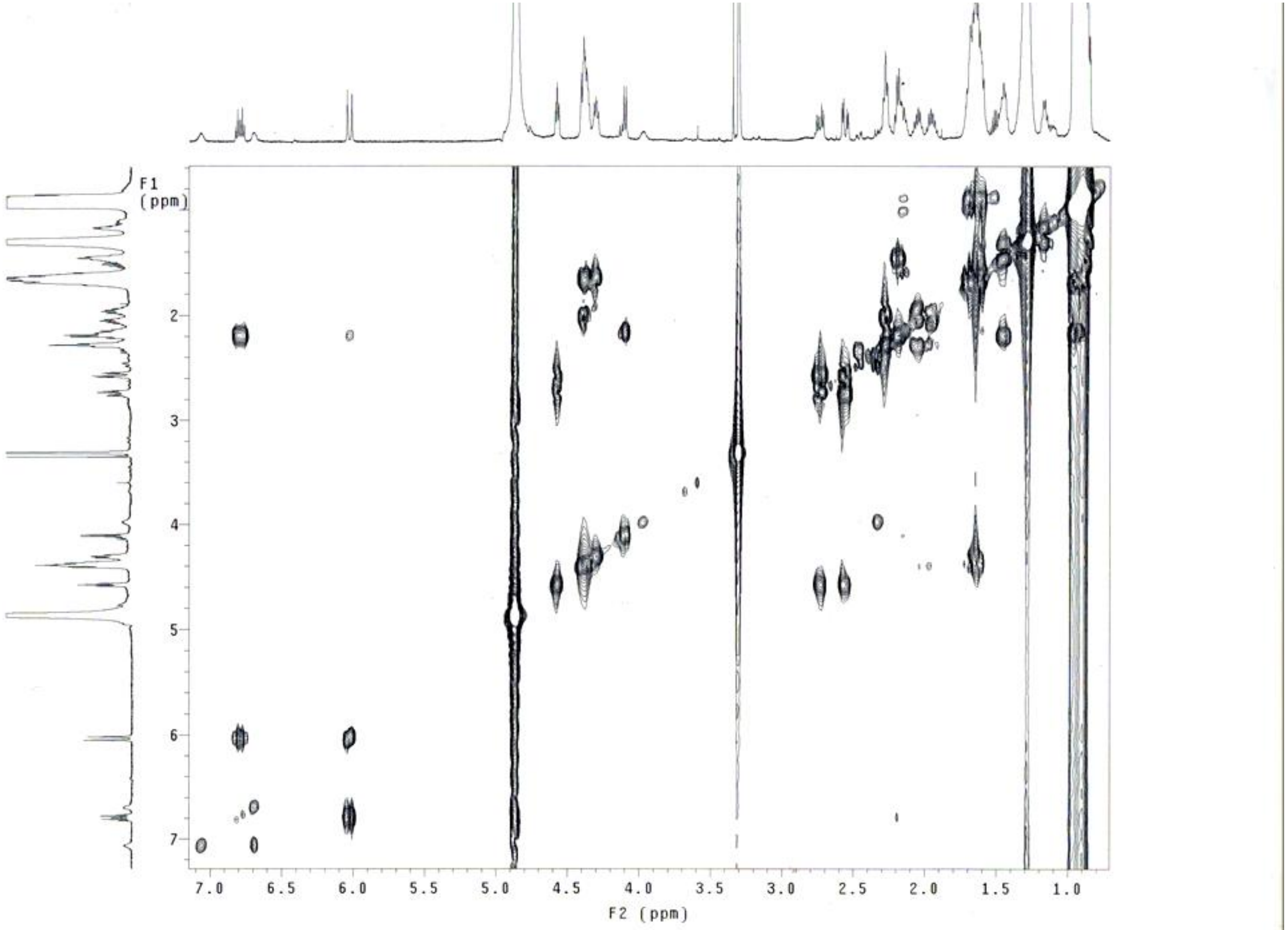

Figure S19. TOCSY spectrum of **3** in CD_3OH.

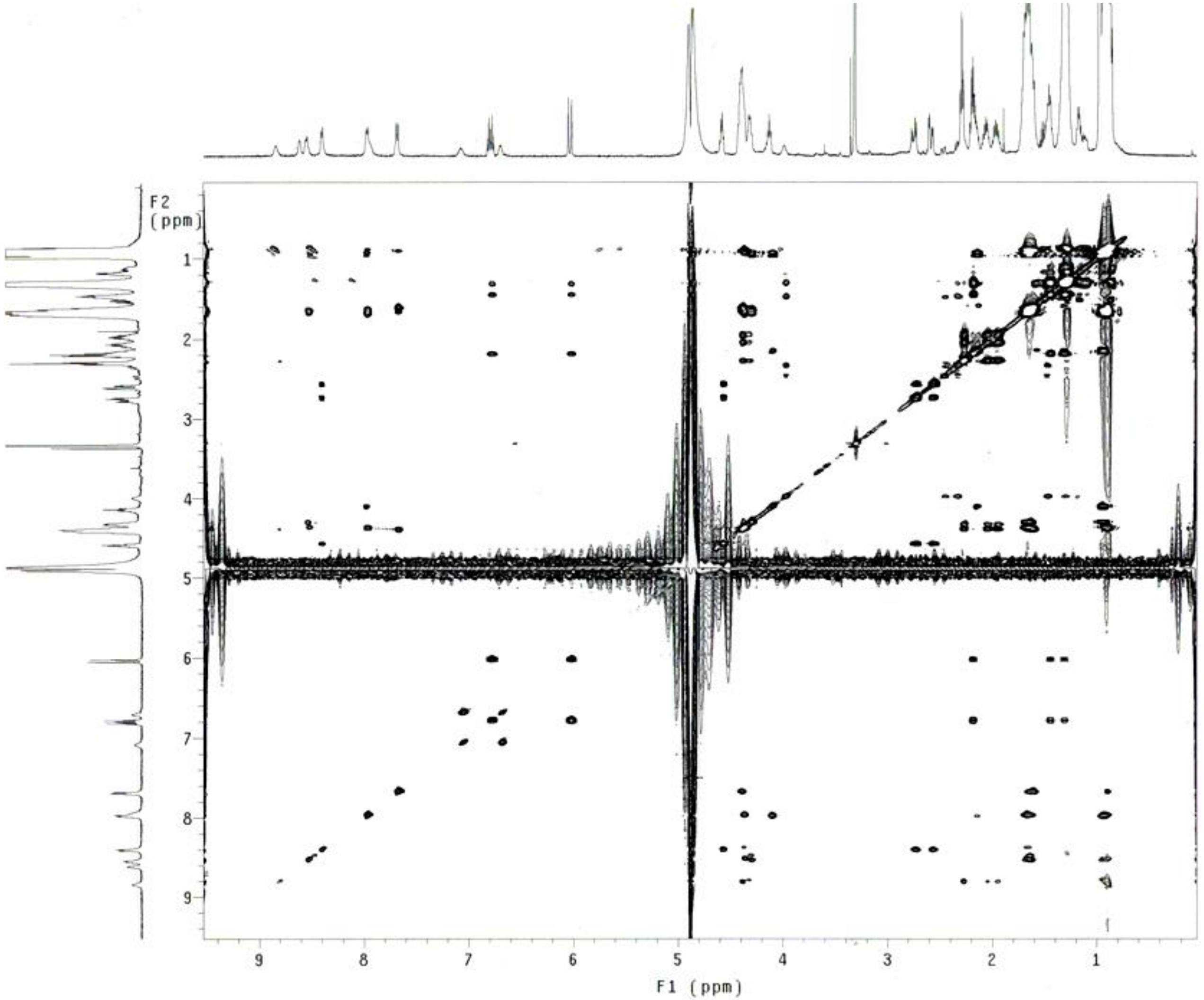

Figure S20. HSQC spectrum of **3** in CD_3OD.

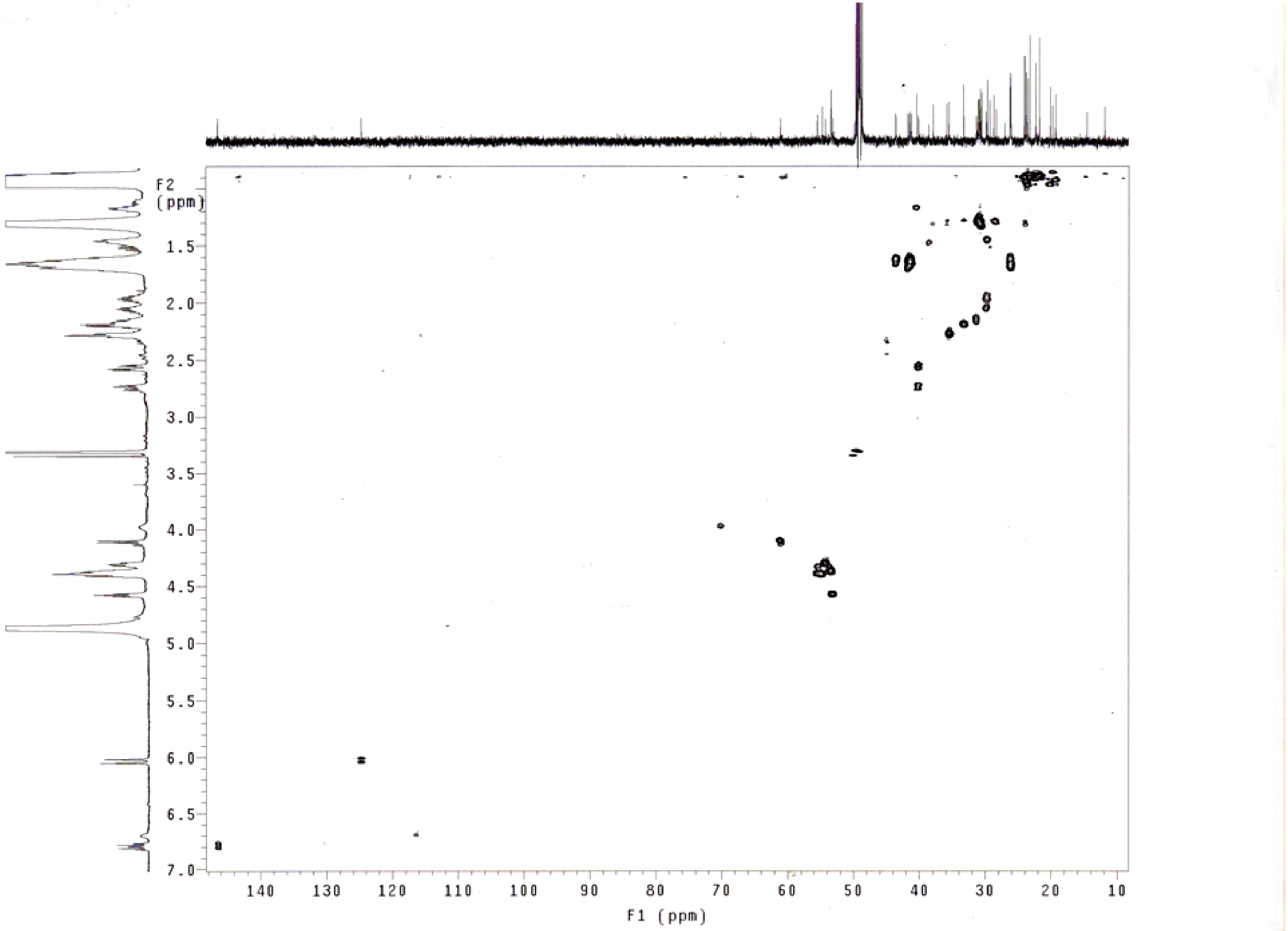

Figure S21. HMBC spectrum of **3** in CD_3OH.

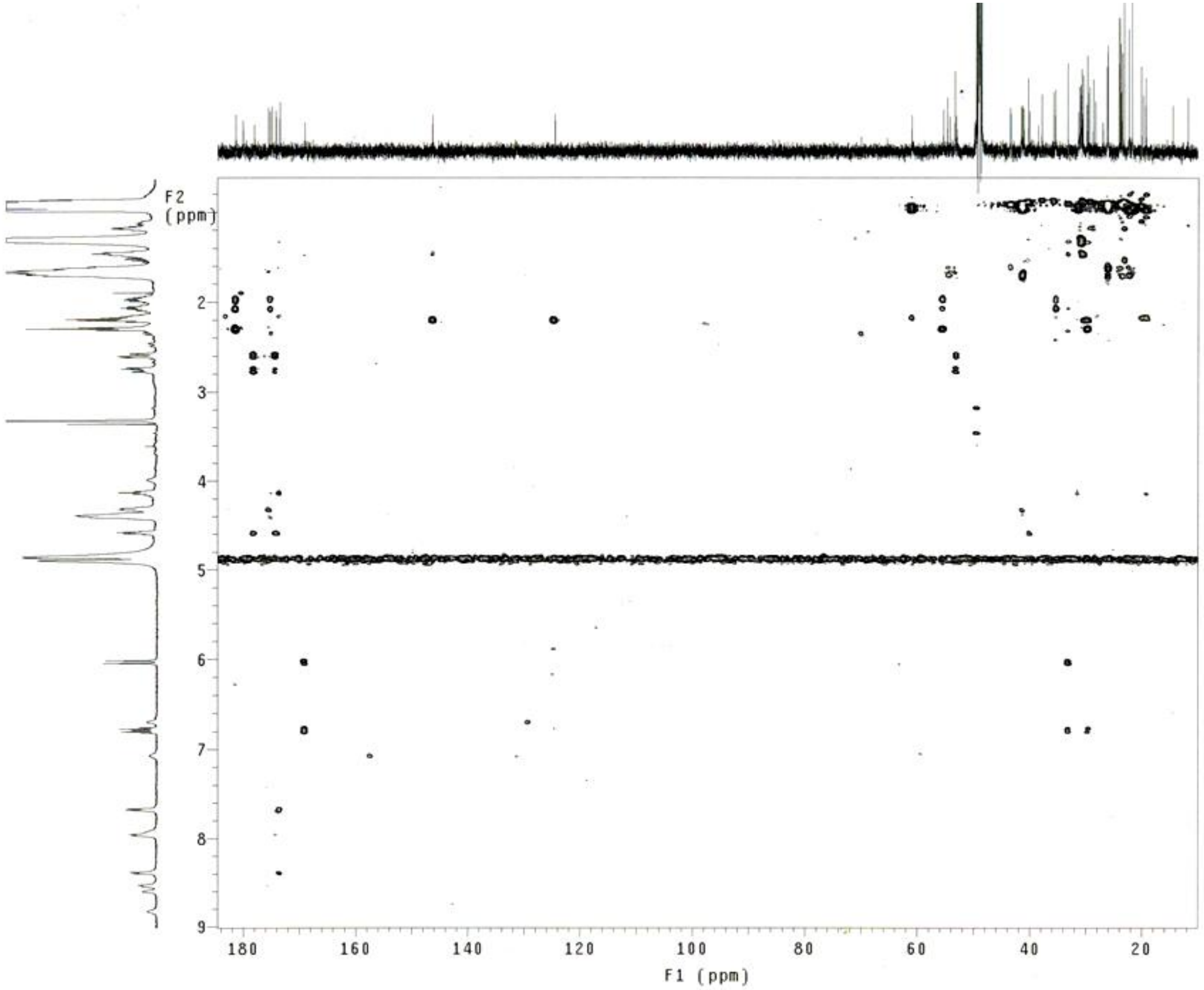

Figure S22. ROESY spectrum of **3** in CD_3OH.

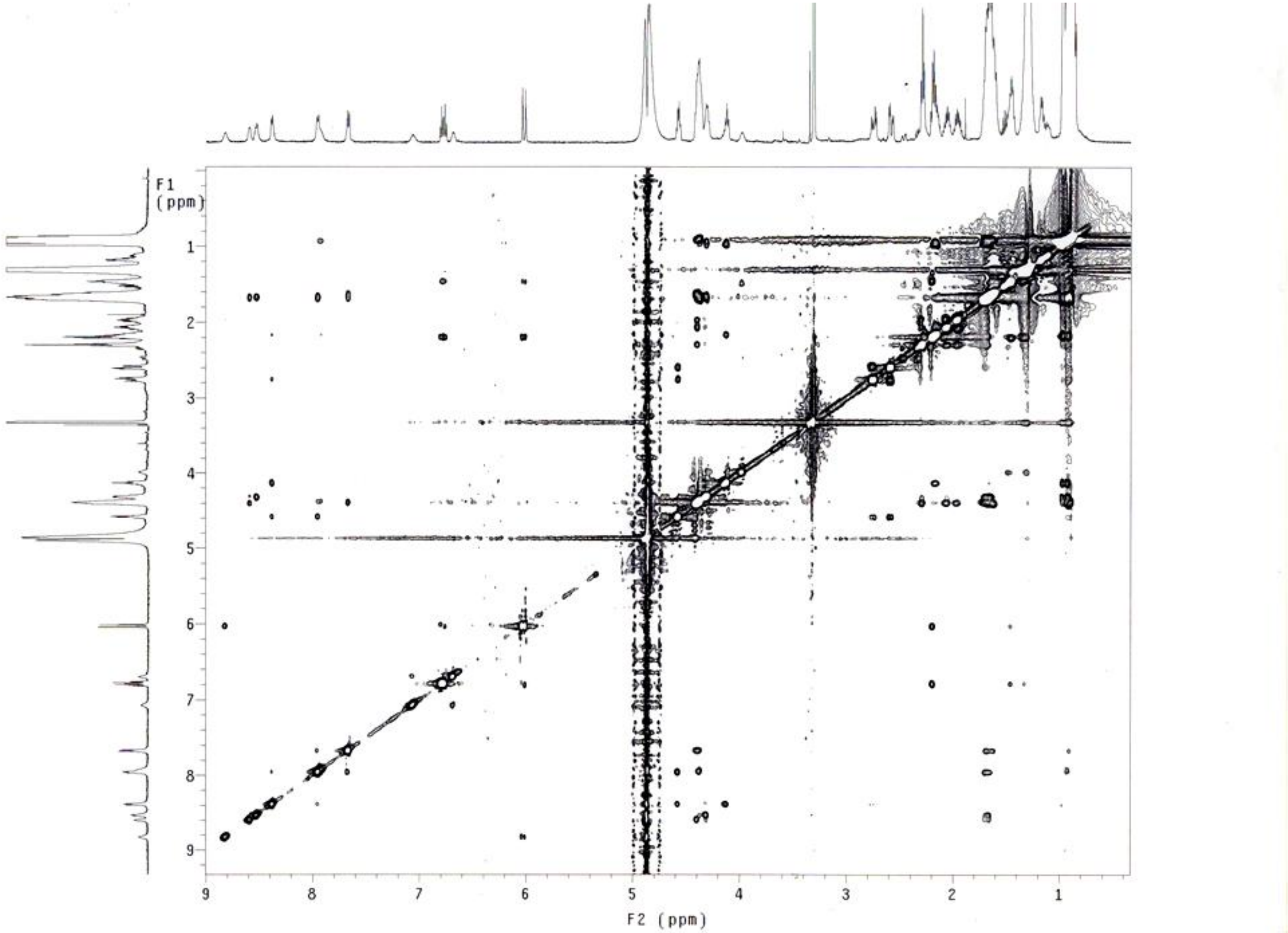

Figure S23. HRESI-MS of **1–3**.

1

2

3

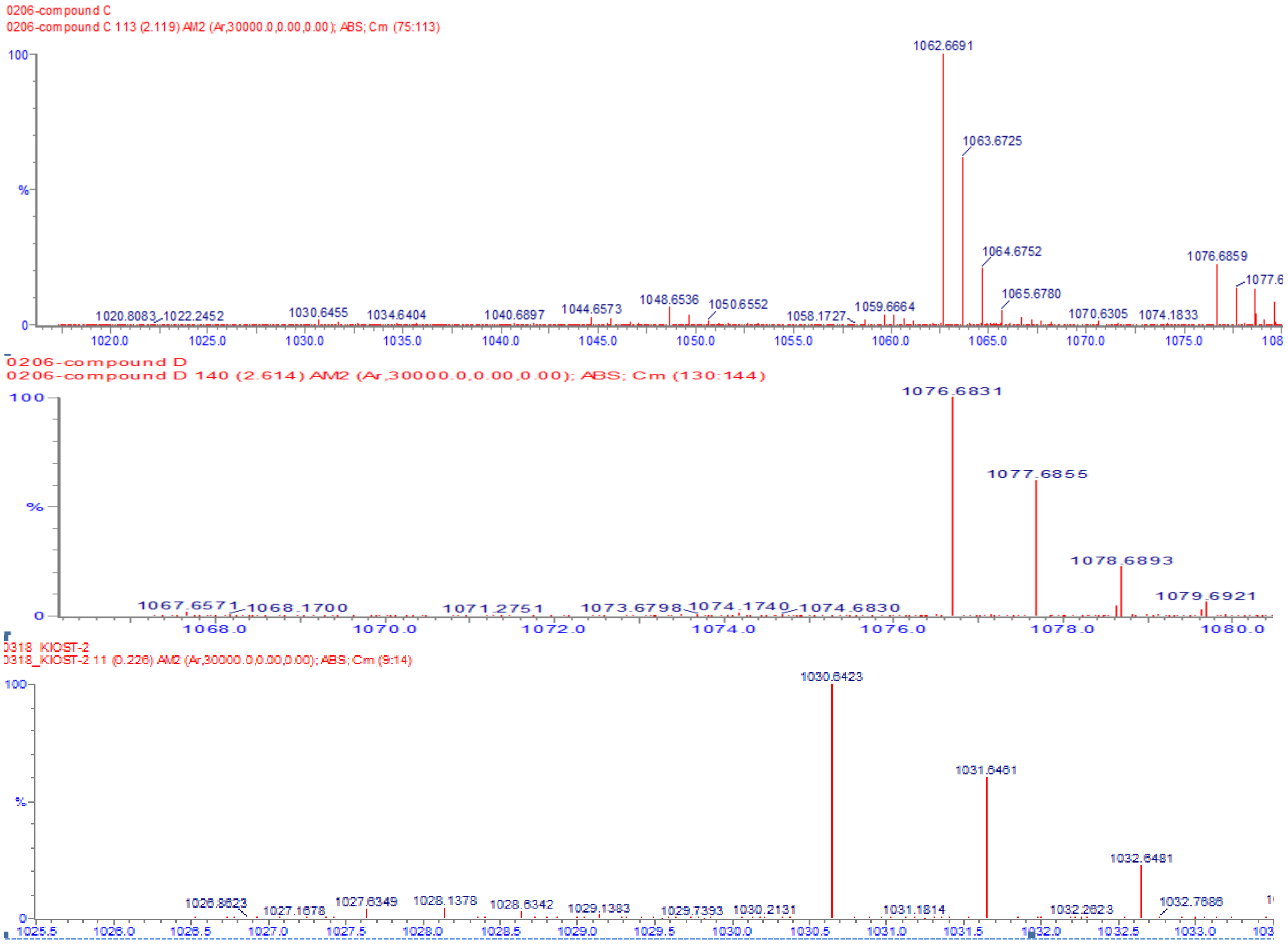

Figure S24. APCI-MS data of fatty acid in **1**.

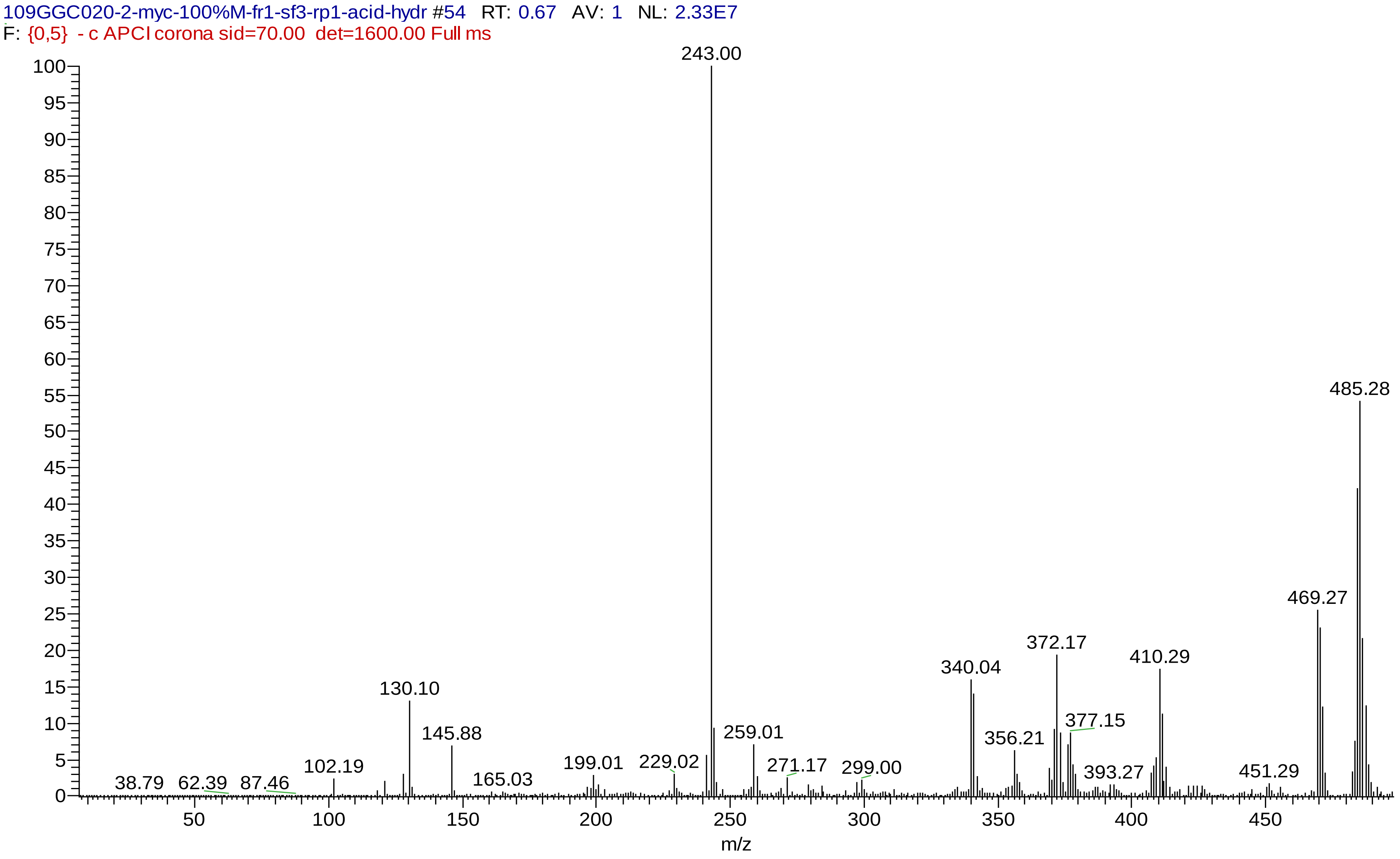

Figure S25. ^{1}H NMR spectrum of fatty acid in **1**.

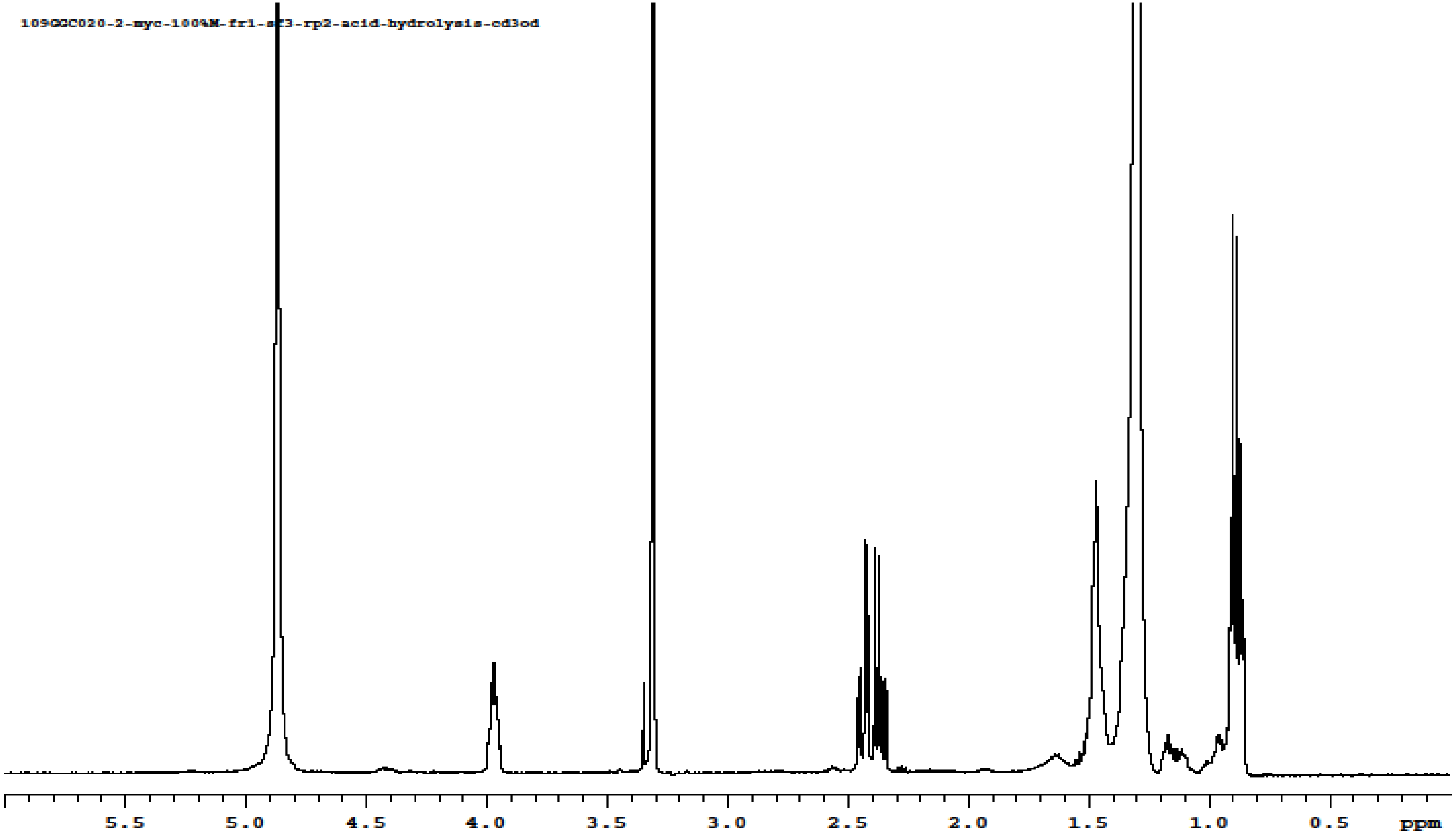

Figure S26. HPLC data for amino acid analysis in **1**.

Gageostatin-A

Solvent system: 40% MeCN in H_2O + 0.5% TFA

Flow- 0.5 ml/min

Injection - 10

Analytical column- C-18

Detector UV, Wavelength -340

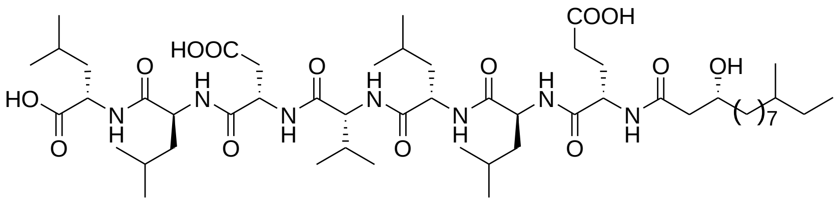

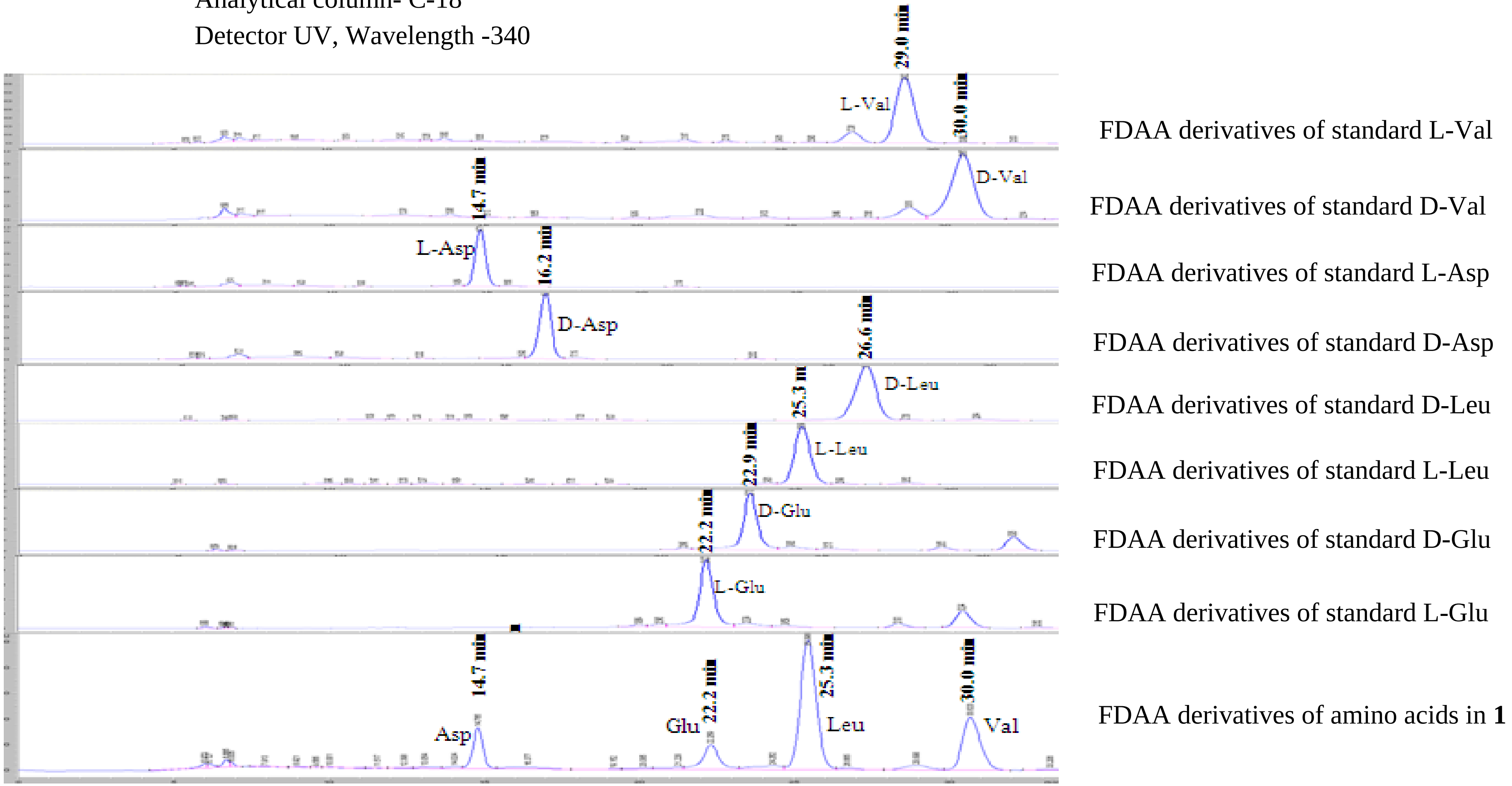

Supplement: Supplementary File 1 — Supplementary Information (PDF, 434 KB) [file marinedrugs-12-00871-s001.pdf]
